# A novel hybrid transformer-based framework (H-ConvNeXt–Swin) to classify brain tumors using MRI

**DOI:** 10.1038/s41598-026-63915-z

**Published:** 2026-08-05

**Authors:** Essam Abdellatef, Rasha M. Al-Makhlasawy, Nesma Abd El-Mawla, Wafaa A. Shalaby

**Affiliations:** 1 Faculty of Computer Science and Engineering, Alamein International University, New Alamein City, Matrouh Egypt 51718; 2 Computers and Systems Department, Electronics Research Institute, Joseph Tito St, El Nozha, P.O. Box: 12622, Cairo, Cairo Governorate Egypt; 3 Department of Computer Science and Engineering, Faculty of Computer Science and Engineering, New Mansoura University, New Mansoura, Egypt; 4 Department of Electronic and Electrical Communication Engineering, Faculty of Electronic Engineering, Menoufia University, Menouf, Egypt

**Keywords:** Deep learning, Artificial intelligence, Convolutional neural networks, Swin transformer, Brain tumor, MRI, Cancer, Computational biology and bioinformatics, Engineering, Health care, Mathematics and computing, Medical research, Oncology

## Abstract

This paper introduces a hybrid deep learning model combining ConvNeXt and Swin Transformer for classifying brain tumors from MRI scans. The ConvNeXt backbone is employed to obtain detailed local spatial features, whereas the Swin Transformer identifies hierarchical long-range dependencies, facilitating complementary feature representation. The proposed model is evaluated on a combined public MRI dataset of 7,023 images distributed across four categories: glioma, meningioma, pituitary, and no tumor. Experimental results demonstrate that the proposed hybrid architecture outperforms several state-of-the-art convolutional and transformer-based models, achieving an accuracy of 95.37% with competitive precision and F-score. Additionally, qualitative explainability assessment using attention-based visualization techniques offers insight into the model’s decision-making by highlighting diagnostically significant regions. Furthermore, we evaluate the proposed H-ConvNeXt-Swin model on the unified dataset and also report source-stratified performance on each of the three constituent datasets: Figshare, SARTAJ, and Br35H. Future work will focus on validating the proposed framework on multi-center clinical datasets and extending it to more complex tasks such as tumor localization and segmentation.

## Introduction

Brain tumors rank among the most aggressive and dangerous neurological conditions, presenting significant difficulties for clinicians because of their varied characteristics and intricate growth behaviors. Precise and prompt identification of brain tumors is essential for establishing suitable treatment plans, forecasting patient results, and enhancing survival rates. Magnetic Resonance Imaging (MRI) continues to be the most commonly utilized non-invasive technique for identifying brain anomalies due to its excellent soft tissue contrast and multiparametric features. However, manually interpreting MRI scans is a demanding job that relies significantly on the skill of radiologists and is susceptible to differences between observers and possible diagnostic mistakes. These constraints emphasize the necessity for computer-aided diagnostic (CAD) systems capable of automatically and accurately classifying brain tumors with high reliability.

In the last ten years, deep learning has become a significant asset in medical image analysis, especially Convolutional Neural Networks (CNNs), which have shown their capacity to autonomously derive hierarchical features from imaging information. Models based on CNN, like VGGNet, ResNet, DenseNet, and EfficientNet, have been extensively utilized in classifying brain tumors and have attained high precision in distinguishing tumor types^1,2^. Even with these developments, CNNs have fundamental constraints in their capacity to grasp long-range dependencies and global context, which are frequently essential in medical imaging applications. Tumor borders and structural patterns can extend over vast areas of the brain, necessitating models that can combine both local and global data.

The rise of Transformer-based architectures in computer vision has introduced a novel approach to tackle these issues. The Vision Transformer (ViT) and its variants like DeiT, Swin Transformer, CvT, and PVT have demonstrated strong results in medical image analysis by utilizing self-attention mechanisms to grasp global dependencies throughout image areas^2–4^. Nevertheless, vanilla ViT models typically necessitate extensive datasets, which are infrequently accessible in the medical field, and might miss the inductive biases essential for grasping detailed local anatomical features. Recent studies, including the Residual Vision Transformer (ResViT) model, utilize self-supervised learning and hybrid CNN–Transformer architectures to address dataset size constraints: in this model, CNNs assist in extracting local features, while Transformer layers capture global patterns; the ResViT method attained better accuracy on public datasets such as BraTS, Figshare, and Kaggle^5^.

To address the shortcomings of both pure CNN and pure Transformer models, recent studies have highlighted hybrid architectures that merge the advantages of both approaches. For instance^6^, presented a hybrid model that merges convolutional layers with transformer components to simultaneously capture local spatial patterns and global context, leading to enhanced classification results. (MDPI) In the study^7^, researchers introduced the SwT+ResNet50V2 model, which combines ResNet50V2 with Swin Transformer. They tackled issues including imaging resolution, class complexity, and training efficiency, attaining high accuracy across both binary and multi-class MRI datasets. A recent study^8^, employed EfficientNetB4 together with transformer blocks, applying methods such as focal loss and test-time augmentation to address class imbalance and minor inter-class variations.

Additionally, sophisticated techniques that tackle data shortages via augmentation or synthetic data have demonstrated potential. In^9^ the authors utilize Autoencoders and cGAN to create synthetic training samples, which helps to address class imbalance and enhance classification accuracy, sensitivity, and specificity on datasets from Figshare and Kaggle. Similarly, CE-RS-SBCIT, launched in 2025, is a hybrid CNN-Transformer that enhances channels with residual, spatial, and boundary-aware learning; this system merges boundary functions and spatial learning within the CNN component along with Transformer modules, demonstrating impressive performance (accuracy ~ 98.30%) on various public MRI tumor datasets^10^. The investigation of self-pretraining and fine-tuning has also been undertaken: the ResViT model integrates CNN and ViT in a self-supervised learning framework, initially training through an MRI synthesis task and subsequently fine-tuning for classification, yielding outstanding accuracy on Figshare and Kaggle datasets^5^. Additionally, there are initiatives to create lightweight or combined models; a deep learning architecture merging Transformers with fusion methods and attention mechanisms (fusion-based deep learning) was suggested in Biomedical Signal Processing & Control (2023), where both local and global features are integrated, employing transfer learning to address overfitting^11^. Furthermore, ResMT presented a hybrid CNN-Transformer model for glioma grading that utilizes 3D MRI, addressing volumetric data and enhancing grading precision despite constrained data availability^12^. Ultimately^13^, evaluates various transformer architectures, such as Swin and MaxViT, across different modalities (MRI, CT), discovering that transformer models generalize effectively to datasets of diverse organs and conditions, highlighting their flexibility in medical imaging applications.

The limitations mentioned indicate that current CNN-based models primarily focus on local texture-level details and consequently find it difficult to depict long-range structural relationships among far-off brain areas in MRI images. In contrast, Transformer architectures excel at global contextual modeling via self-attention, yet their performance declines on smaller and more varied medical datasets because of a deficiency in convolutional inductive biases and their considerable data dependency. This trade-off suggests that neither CNNs nor standalone Transformers offer a well-rounded representation for classifying brain tumor MRIs. Inspired by this finding, the suggested framework combines a ConvNeXt stem to maintain high-resolution tissue-level structural information with Swin Transformer blocks that collect hierarchical long-range dependencies via shifted-window attention. This hybrid approach addresses the previously recognized methodological shortcomings by integrating complementary representation methods within a cohesive framework, thus increasing resilience to MRI variability and boosting tumor differentiation.

Even though numerous CNN and Transformer architectures have been suggested for classifying brain tumor MRIs, existing approaches continue to face two primary limitations. Initially, CNN models primarily acquire localized feature representations and thus struggle to effectively capture long-range contextual relationships that are crucial for distinguishing tumor boundaries and global anatomical features. Secondly, pure Transformer architectures depend significantly on extensive training datasets and lack convolutional inductive biases, which diminishes their stability and generalization when utilized with varied medical images and limited clinical datasets. Consequently, current methods either deliver robust local feature representation with minimal contextual understanding, or they seize global interrelationships at the cost of resilience and data efficiency. This study fills this void by creating a hybrid architecture that integrates ConvNeXt-based convolutional feature extraction with the hierarchical shifted-window attention from Swin Transformer, aiming to produce a balanced representation that simultaneously captures local tissue features and global structural relationships in brain MRI images.

In this study, we present a hybrid framework that integrates ConvNeXt, a contemporary CNN backbone, with Swin Transformer to classify brain tumors using MRI images. Our goal is to utilize local feature extraction alongside global contextual modeling in a cohesive framework. The suggested model is assessed using a publicly accessible aggregated dataset and benchmarked against various leading-edge methods. Although the framework shows impressive classification results, the present research is confined to image-level classification and does not tackle pixel-level tasks like tumor localization or segmentation. These elements are viewed as components of upcoming tasks. The primary contributions of this paper are outlined as follows:

1. A hybrid deep learning model (H-ConvNeXt–Swin) that combines convolutional feature extraction with hierarchical transformer attention for classifying brain tumors.
2. An extensive experimental assessment on a combined public MRI dataset, showing enhanced classification results in comparison to various CNN and transformer benchmarks.
3. A qualitative insights analysis utilizing attention-based visualization methods to clarify model decision-making.
4. A structured comparison with leading architectures backed by statistical assessment and performance measures.
5. A conversation about constraints and future pathways for enhancing generalization and broadening the framework to encompass more sophisticated medical imaging challenges.

The remainder of this paper is organized as follows: literature review of brain tumors and deep learning are discussed in section two. On the other hand, section three is concerned with the description of the proposed framework. Section four explored and discussed the experimental results among real-world dataset. Finally, the paper is concluded in section five.

## Related work

Initial efforts in classifying brain tumors largely utilized conventional machine learning techniques that were based on the extraction of features manually crafted. Texture descriptors, shape characteristics, and intensity histograms were obtained from MRI scans and subsequently classified with algorithms including Support Vector Machines (SVM), k-Nearest Neighbors (k-NN), and Random Forests. Although these approaches reached moderate effectiveness, they relied heavily on domain-specific feature engineering and did not demonstrate generalizability across various datasets. Consequently, they were slowly substituted by deep learning methods that provided comprehensive feature extraction and classification.

Convolutional Neural Networks (CNNs) have been pivotal in enhancing brain tumor classification. Numerous studies have shown the efficiency of CNN in attaining high diagnostic precision. For example^1^, utilized ResNet for classifying brain tumors and achieved accuracies exceeding 94% on the Figshare dataset, emphasizing the efficiency of transfer learning in medical imaging. In the same vein^2^, utilized DenseNet201 for MRI classification, attaining performance above 95%, highlighting how densely connected layers can improve feature reuse and gradient flow. In^14^, the authors present super-resolution and image fusion using CNN techniques, attaining an accuracy of approximately 99.74% on publicly available 2D MRI datasets, illustrating that CNNs can remain highly efficient even without transformer modules when integrated with preprocessing and fusion methods. Nonetheless, CNNs are constrained by their local receptive fields, which restrict their capacity to represent global contextual dependencies in brain images.

To tackle these problems, Transformer-based models have been implemented in medical imaging. The Vision Transformer (ViT) showcased the potential of utilizing self-attention mechanisms for visual tasks and was subsequently employed in brain tumor classification, yielding encouraging outcomes^15^. In^16^, various ViT variants (including ViT-b16, ViT-l16, etc.) were fine-tuned using brain tumor MRI images; findings showed that larger ViTs provide better performance but require more computational resources and data. Furthermore, within multi-organ and multimodal contexts, the research titled^17^ evaluated Swin Transformer and MaxViT using MRI and CT datasets, demonstrating Swin’s strength particularly in heterogeneous data situations. (arXiv) Self-supervised Transformer methods, like ResViT, have enhanced performance by being pretrained on MRI synthesis tasks prior to classification to supplement constrained real-data situations.

Even with the advancements of Transformers, their dependence on extensive datasets and absence of inductive biases prompted the creation of hybrid CNN–Transformer structures. These models merge the local feature extraction strengths of CNNs with the global self-attention features of Transformers to attain enhanced performance. For instance^6^, presented a model that merges convolutional layers with transformer components, demonstrating superior accuracy compared to CNN-only baselines. In^18^, the SwT+ResNet50V2 framework merged feature extraction through ResNet50V2 with Swin’s self-attention, attaining almost flawless performance in binary classification and impressive outcomes in four-class classification. In a similar vein, CE-RS-SBCIT suggested a channel-enhanced hybrid design featuring spatial and boundary-aware components along with hybrid transformer blocks, achieving approximately 98.30% accuracy. Hybrid models that utilize data augmentation or synthetic data, such as the GAN + Autoencoder + Swin Transformer projects from 2025, demonstrate that the integration of data generation and transformer classification greatly boosts generalization, sensitivity, and specificity^9^. Moreover, in ResMT, a hybrid CNN-Transformer model was employed for grading volumetric gliomas using 3D MRI, which is important as volumetric data introduces extra spatial complexity; nevertheless, the model achieved state-of-the-art performance^12^. Additional research such as^19^ integrates CNN + Transformer while also tackling class imbalance and nuanced inter-class differences through contemporary loss functions and test-time augmentation. (JISEM) In conclusion^20^, combines attention and cross-fusion techniques with lightweight CNNs (iResNet, etc.), demonstrating that an appropriately structured fusion of local and global features can decrease overfitting and enhance performance on varied MRI datasets.

Reference^20^ Presented a recent and pertinent CAD study for classifying brain tumors, where the authors introduced a CNN-based transfer-learning framework that integrates comprehensive preprocessing tasks such as image resizing, normalization, object-centric extraction, and contrast enhancement, and assesses performance across axial, sagittal, and coronal MRI perspectives. Their model attained impressive accuracy in multi-class classification and notable AUC, precision, and F1-score, showcasing the efficacy of CNN-based feature learning coupled with multi-view MRI integration. Nevertheless, despite enhancing feature-level discriminative ability, this method is constrained to convolutional representations and fails to explicitly capture long-range contextual dependencies or hierarchical structural relationships among brain regions, which are crucial for effective tumor differentiation in heterogeneous datasets. Conversely, the suggested hybrid ConvNeXt–Swin Transformer framework aims to overcome this constraint by simultaneously capturing local tissue-level signals and global contextual relationships via the combination of convolutional inductive biases and hierarchical shifted-window self-attention.

Recent research^21^ explores enhanced convolutional methods, focusing on improved convolutional learning strategies and sophisticated augmentation workflows designed specifically for brain MRI. This research enhances the comprehension of how modifications in architecture and refinements in training can boost performance; however, like other CNN-based methods, it fails to directly tackle the requirement for modeling long-range contextual relationships within spatially extensive tumor structures.

Reference^22^ Recently assessed a Vision Transformer-based model for classifying and predicting brain tumors from clinical MRI data, demonstrating encouraging outcomes in detecting tumor subtypes with notable sensitivity and specificity. Their research emphasizes the promise of self-attention mechanisms in clinical imaging applications while also revealing typical challenges in data preprocessing and model optimization for limited medical datasets. This additionally reinforces our focus on hybrid frameworks that integrate convolutional inductive biases with hierarchical attention to enhance representation consistency .

Previous deep learning frameworks for brain tumor classification, like the research presented at^22^, made significant contributions by showcasing the viability of convolutional feature learning for differentiating between multiple tumor classes. Although these early studies showed encouraging outcomes, they were constrained in scope and did not incorporate advanced architectural features for capturing hierarchical and global context, a limitation that future transformer and hybrid models seek to address. Table 2 illustrates an analytical comparison of recent brain tumor MRI classification studies. Table 1 illustrates a comparative summary of related works.

**Table 1:** Analytical comparison of recent brain tumor MRI classification studies.

Reference	Model type	Dataset	Modality	Reported accuracy (%)	Other metrics (reported)	Key limitations
^13^	CNN-based	Private MRI	MRI (2D)	~ 91.0	Precision, Recall	Early CNN; limited data and no global context
^1^	ResNet + TL	Figshare	MRI (2D)	94.0	Precision, Recall, F1	CNN captures local features only
^2^	DenseNet201	Private MRI	MRI (2D)	95.0	Accuracy	No attention or global modeling
^4^	Vanilla ViT	BraTS	MRI (2D)	88.0	Accuracy	Requires large-scale data
^14^	DeiT	Figshare	MRI (2D)	92.0	F1-score	Limited local inductive bias
^7^	Swin Transformer	BraTS	MRI (2D/3D slices)	94.5	AUC ≈ 0.98	Computationally expensive
^21^	Enhanced CNN	Public MRI	MRI (2D)	96.0	Accuracy	CNN-only representation
^22^	Hybrid CNN + Attention	Public MRI	MRI (multi-modal)	97.2	F1, AUC	Late-stage feature fusion
^20^	CNN + Transfer Learning	Kaggle, Figshare	MRI (Axial/Sagittal/Coronal)	98.5	Precision, Recall, F1, AUC	No explicit long-range attention
**Proposed (this work)**	**ConvNeXt + Swin**	**Kaggle MRI**	**MRI (2D)**	**95.37**	**Precision**,** Recall**,** F1**,** AUC**	**Balanced local & global modeling**

Despite the impressive advancements in brain tumor classification shown by the aforementioned studies utilizing CNN-based, Transformer-based, and hybrid architectures, many of them still face limitations in at least one of the following aspects. Initially, CNN-based methods mainly emphasize localized texture and intensity characteristics, lacking an explicit model for long-range contextual dependencies between spatially remote brain regions. Secondly, purely Transformer-based models excel at global feature modeling but necessitate extensive training datasets and are devoid of convolutional inductive biases, which diminish their robustness in diverse and limited medical datasets. Third, numerous recent CAD frameworks driven by hybrid and transfer learning achieve high accuracy; however, they primarily depend on improved preprocessing and feature-level fusion instead of explicitly combining complementary local and global representation mechanisms in a cohesive architecture. These constraints create a need for a hybrid framework that simultaneously maintains detailed local tissue characteristics while recognizing hierarchical global interconnections, which forms the primary design goal of the ConvNeXt–Swin Transformer model proposed in this research.

## Proposed framework

**Fig. 1:**
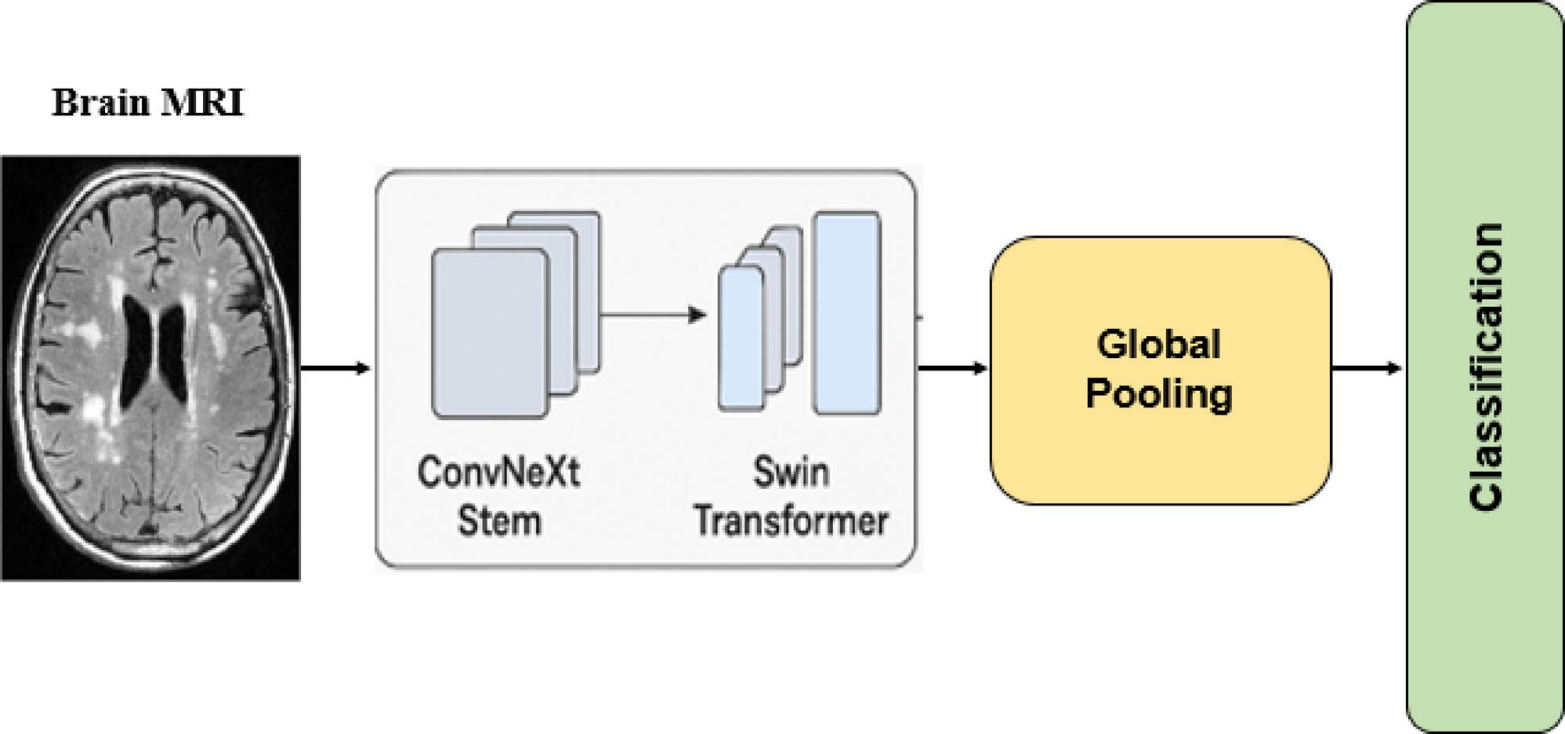
Proposed hybrid framework for brain tumor classification.

The proposed framework (H-ConvNeXt–Swin) applies a hybrid deep learning model to classify brain tumors from MRI scans, as shown in Fig. 1. It starts with a ConvNeXt stem that learns low-level spatial representations such as edges, textures, and local anatomical structures. After that, these features are passed through Swin Transformer blocks that refine them by modeling both local dependencies within image regions and long-range contextual relationships across the brain volume. The hierarchical shifted-window mechanism of Swin enables efficient feature aggregation at multiple scales. After feature extraction, a global pooling layer condenses the multi-dimensional representation into a compact vector, which is finally fed into a classification step that predicts the tumor category. This integration of convolutional inductive biases with transformer attention ensures that both local tissue characteristics and global structural patterns are effectively represented. To evaluate performance, we will use accuracy, precision, and F-score, along with ROC curves and confusion matrices, ensuring both numerical and visual insights into the model’s effectiveness.

### Stages of H-ConvNeXt–Swin framework

The Proposed hybrid framework for brain tumor classification has key four stages. It illustrates a deep learning framework designed to take a brain scan MRI image as input and output a classification result, likely indicating the presence of a brain tumor. Next sub-sections illustrate each stage of the proposed model. In Table 2, The proposed H-ConvNeXt–Swin framework adopts a sequential–parallel hybrid integration strategy that combines the strengths of convolutional and transformer-based architectures. Specifically, ConvNeXt-Tiny, consisting of approximately 28 M parameters, is employed in the early stages to extract fine-grained local spatial features through its first two blocks. The resulting feature maps of size 28 × 28 × 192 are subsequently projected to 96 channels via a 1 × 1 convolution followed by layer normalization to ensure dimensional compatibility with the Swin-Tiny transformer backbone. Swin-Tiny, comprising approximately 29 M parameters with a window size of 7 and a patch size of 4, is then utilized to capture long-range contextual dependencies. Parallel fusion is performed from Stage 2 onward using a multi-head cross-attention mechanism with four attention heads at a spatial resolution of 14 × 14, enabling effective interaction between convolutional and transformer features. The fused representations are refined through residual connections and concatenation after channel alignment. Finally, a lightweight classification head, consisting of global average pooling, layer normalization, dropout with a rate of 0.2, and a fully connected layer mapping 256 features to four output classes, is employed to generate the final predictions.

**Table 2:** Architectural configuration and key implementation details of the proposed hybrid H-ConvNeXt–Swin framework.

Component	Specification	Parameters	Output Size	Channels
Input	224 × 224 × 3	–	224 × 224	3
ConvNeXt stem	ConvNeXt-Tiny Block 1	28 M	56 × 56	96
	ConvNeXt-Tiny Block 2	–	28 × 28	192
Feature interface	1 × 1 Conv + LayerNorm	0.4 M	28 × 28	192→96
Swin transformer	Swin-Tiny Stage 1	29 M	28 × 28	96
	Swin-Tiny Stage 2	–	14 × 14	192
	Swin-Tiny Stage 3	–	7 × 7	384
	Swin-Tiny Stage 4	–	7 × 7	768
Fusion mechanism	Parallel attention-based fusion at Stage 2	1.2 M	14 × 14	192 + 192→256
Classifier head	Global Avg Pool → LayerNorm → Dropout(0.2) → FC	0.8 M	1 × 1	256→4
Total parameters	–	59.4 M	–	–

#### Stage 1 - input

The process within the system begins with the Brain image, i.e., the MRI image (Magnetic Resonance Imaging) of the human brain. The input image to highlight lesions, i.e., the bright hyperintense areas visible within the image, which could be potential tumors. This is the raw data on which the system will be working.

#### Stage 2 - feature extraction: hybrid deep learning model

The core of the system is the hybrid deep learning structure responsible for extracting informative features from the brain image. This component is further broken down into two consecutive sub-parts:

- ConvNeXt Stem because it is one of the state-of-the-art CNN architectures distinguished by high performance that usually borrows the design principles from Vision Transformers but still inherits the effectiveness of CNNs. Typically, the Stem is about the early layers within the network where the first few steps of the convolution, downsampling, and first generation of the feature maps take place. Here, its role is to handle the raw image pixels and produce the first few low-level to mid-level representations of the features.
- Swin Transformer because it is a Vision Transformer that employs shifted windows to implement self-attention. This architecture greatly enhances computational efficiency and enables the model to efficiently handle local details (inside one window) and global contextual information (over shifted windows) and is hence equally applicable to high-resolution image tasks such as medical image analysis. This section receives the features output by the ConvNeXt Stem and processes them with the strong, contextual attention mechanisms within the Swin Transformer.

Lastly, the integration of ConvNeXt to localize efficiently and Swin Transformer to model strong long-range dependency is the reason this is referred to as a hybrid framework.

#### Stage 3 - dimensionality reduction: global pooling

Once the hybrid model has retrieved a set of strong features, the output goes to a Global Pooling layer. Global pooling is an operation that decreases the spatial dimensions (width and height) of the feature maps. Global pooling narrows down each of the feature maps to one value. What is left is the fixed-length feature vector that encapsulates the strongest features learned by the entire image and is ready to be used by the final classification step.

#### Stage 4 - output

The final layer is the Classification layer (a fully connected layer with softmax activation). This is the layer to which the globally-pooled feature vector from the Global Pooling step is input. They output the probability scores of each potential class. The final output is the prediction of the system. Overall, the system utilizes an exemplary hybrid architecture (ConvNeXt + Swin Transformer) to scrutinize brain MRI images, efficiently discerns key characteristics, abstracts them through Global Pooling, and subsequently renders an unequivocal Classification judgment on the brain tumor.

### Core mathematical concept of H-ConvNeXt–Swin framework

In contrast to benchmark architectures like DeiT, PiT, PvT, CvT, and T2T-ViT that are only used for comparative analysis, the suggested framework is composed solely of ConvNeXt and Swin Transformer components. Consequently, the mathematical formulation provided here centers exclusively on the applied H-ConvNeXt–Swin architecture.Represent the input MRI image as:

$$\:\mathrm{X}\:\in\:{\mathbb{R}}^{\mathrm{H}\:\times\:\mathrm{W}\:\times\:\mathrm{C}}$$

where $\:\mathrm{H},\:\mathrm{W}$, and $\:\mathrm{C}$ represent the height, width, and count of channels in the image, respectively.

The ConvNeXt backbone is initially used to derive local spatial features from the input image via depthwise convolution techniques. The related operation can be stated as:

$$\:\mathrm{Y}\:=\:\mathrm{D}\mathrm{W}\mathrm{C}\mathrm{o}\mathrm{n}\mathrm{v}\left(\mathrm{X}\right)$$

where $\:\mathrm{D}\mathrm{W}\mathrm{C}\mathrm{o}\mathrm{n}\mathrm{v}\left(\mathrm{X}\right)$ signifies the depthwise convolution operation and $\:\mathrm{Y}$ denotes the extracted local feature maps.

The created feature maps are subsequently projected and sent to the Swin Transformer module. For each local window, the computation of the self-attention operation is performed as follows:

$$\:\mathrm{A}\mathrm{t}\mathrm{t}\mathrm{e}\mathrm{n}\mathrm{t}\mathrm{i}\mathrm{o}\mathrm{n}\left(\mathrm{Q},\mathrm{K},\mathrm{V}\right)=\mathrm{S}\mathrm{o}\mathrm{f}\mathrm{t}\mathrm{m}\mathrm{a}\mathrm{x}\left(\frac{\mathrm{Q}{\mathrm{K}}^{\mathrm{T}}}{\sqrt{\mathrm{d}}}\right)\mathrm{V}$$

where $\:\mathrm{Q},\mathrm{K}$, and $\:\mathrm{V}$ stand for the query, key, and value matrices, respectively, and d indicates the feature dimension.

To enable information transfer among adjacent windows, the shifted-window multi-head self-attention mechanism is utilized as:

$$\:{\mathrm{Z}}^{\mathrm{l}}=\:\mathrm{S}\mathrm{W}-\mathrm{M}\mathrm{S}\mathrm{A}\left({\mathrm{Z}}^{\left\{\mathrm{l}-1\right\}}\right)$$

where $\:{\mathrm{Z}}^{\mathrm{l}}$ indicates the output feature representation at layer $\:\mathrm{l}$, and $\:\mathrm{S}\mathrm{W}-\mathrm{M}\mathrm{S}\mathrm{A}$ refers to the shifted-window self-attention process.

Following the hierarchical feature extraction, global average pooling is utilized to achieve a concise feature representation:

$$\:\mathrm{f}\:=\:\mathrm{G}\mathrm{A}\mathrm{P}\left(\mathrm{Z}\right)$$

where $\:\mathrm{G}\mathrm{A}\mathrm{P}\left(\mathrm{Z}\right)$ represents the operation of global average pooling.

Ultimately, the resulting feature vector is sent to the classification layer to produce the probability distribution for the four tumor types:

$$\:\widehat{\mathrm{y}}\:=\:\mathrm{S}\mathrm{o}\mathrm{f}\mathrm{t}\mathrm{m}\mathrm{a}\mathrm{x}\left(\mathrm{W}\mathrm{f}+\mathrm{b}\right)$$

where $\:\mathrm{W}$ and $\:\mathrm{b}$ represent the adjustable weight matrix and bias vector, respectively, and $\:\widehat{\mathrm{y}}$ indicates the predicted class probabilities.

The mathematical formulation mentioned above illustrates the real computational process utilized in the proposed H-ConvNeXt–Swin framework and distinctly separates it from the benchmark transformer models that are only employed for performance evaluation in the experimental analysis.

Table 3 showcases the mathematical formulation of the components incorporated in the proposed H-ConvNeXt–Swin framework. In contrast to the benchmark architectures covered in the literature, the proposed model includes only ConvNeXt and Swin Transformer. ConvNeXt enables effective local feature extraction via convolutional inductive biases, while Swin Transformer captures hierarchical long-range dependencies through shifted-window self-attention. The outcomes of feature representations are compressed through global average pooling and categorized using a Softmax layer. This integration enables the framework to efficiently utilize both local and global data present in brain MRI images.

**Table 3:** Mathematical formulation of the main components in the proposed H-ConvNeXt–Swin architecture.

Component	Mathematical operation	Purpose
ConvNeXt	Depthwise convolution	Local feature extraction
Swin transformer	Window-based Self-Attention	Global contextual modeling
SW-MSA	Shifted window attention	Cross-window interaction
Global pooling	GAP	Dimensionality reduction
Softmax classifier	FC + Softmax	Tumor classification

## Experimental results

A novel MRI classification model for brain tumours has been put forth. Accurately identifying high-performance MRI images is the main goal of the suggested paradigm. Several experiments were carried out in order to evaluate and test the proposed model and its phases. The first set of tests evaluated the structural efficacy of the suggested model and examined how it affected classification performance. The second set of tests evaluated the general structure of the proposed model and contrasted it with state-of-the-art algorithms.

### Dataset description and performance evaluation metrics

The experiments was carried out on Brain Tumor MRI Dataset. It is the union of the following three datasets: figshare, SARTAJ dataset, and Br35. The data contain 7023 human brain MRI image data that are classified into 4 classes: glioma - meningioma - no tumor and pituitary. No tumor class images were sampled from the Br35H image dataset.

In this study, the model’s performance is evaluated using Accuracy, Precision, and F1 Score. The accuracy of BC image recognition is provided by Eq. (1). Equation (2) illustrates how the ratio of correctly detected images was determined using precision. Equation (3) illustrates the relationship between the F1-score, which represents precision and recall. It is a trustworthy indicator of unbalanced data.

1 $$\:\mathrm{Accuracy}=\frac{\mathrm{TP+TN}}{\mathrm{FN+TN+TP+FP}}$$

2 $$\:\mathrm{Precision}\text{}\mathrm{=}\text{}\frac{\mathrm{TP}}{\mathrm{FP+TP}}\:$$

3 $${\text{F1 - score = }}~2~ \times ~\left( {\frac{{{\mathrm{Recall}} \times {\mathrm{Precision}}}}{{{\mathrm{Recall}}~ + ~{\mathrm{Precision}}}}} \right)$$

Where TN, FN, FP, and TP refer to “true negative,” “false negative,” “false positive,” and “true positive,” respectively.

**Table 4:** Training Protocol and Hyper-parameter Configuration of the Proposed H-ConvNeXt–Swing framework.

Feature	Description
Total images	7,023 MRI images
Number of classes	4 (Glioma, Meningioma, Pituitary, No Tumor)
Input preprocessing	2D MRI slices as provided in the Kaggle dataset; resized to 224 × 224 pixels using bilinear interpolation; grayscale images replicated to 3 channels to match model input requirements (224 × 224 × 3)
Data augmentation (50% probability each)	Random rotation (± 15°); horizontal flip (50%); affine transformation: scale (0.9–1.1), translation (± 10%); brightness/contrast adjustment (± 0.1); gamma correction (γ ∈ [0.7, 1.3])
Optimizer	AdamW (β₁=0.9, β₂=0.999)
Initial learning rate	1 × 10⁻⁴
Learning rate schedule	Cosine annealing with warm restarts (T₀=30, T_mult = 2, η_min = 1 × 10⁻⁶)
Warmup strategy	Linear warmup for first 5 epochs
Batch size	16 per GPU (effective batch size = 32 using gradient accumulation)
Total epochs	120
Early stopping	Patience = 15 epochs (based on validation macro F1-score)
Weight decay	0.05
Primary loss function	Focal Loss (γ = 2.0)
Class weights	[0.25, 0.30, 0.20, 0.25] for [Glioma, Meningioma, No Tumor, Pituitary]
Initialization	ConvNeXt and Swin blocks pretrained on ImageNet-22 K; fusion layers initialized using He normal; classifier head using Kaiming uniform
Training strategy	End-to-end fine-tuning with discriminative learning rates
Regularization	Dropout: 0.1 after attention, 0.2 before classifier; stochastic depth: 0.1
Validation protocol	5-fold cross-validation with stratified splitting (train/validation/test performed within each fold)
Ensemble strategy	Weighted averaging of top-3 checkpoints
Hardware & software	2× NVIDIA RTX 3090 (24GB); PyTorch 2.0; mixed precision (AMP)
Training time	~ 18 h

Table 4 summarizes the complete training protocol and hyper-parameter configuration of the proposed H-ConvNeXt–Swin framework. The model operates on 2D MRI slices as provided in the Kaggle dataset, where each slice is treated as an independent sample. All images are resized to 224 × 224 pixels and converted to 3-channel inputs by channel replication to match the network architecture requirements. Data augmentation strategies are applied to improve generalization and robustness. The model is optimized using AdamW with a cosine annealing learning rate schedule and trained end-to-end with discriminative fine-tuning. Regularization techniques, including dropout and stochastic depth, are employed to reduce overfitting. Additionally, focal loss with class weights is used to address class imbalance. This well-defined pipeline ensures reproducibility, stable convergence, and fair performance evaluation.

### Explainable AI and visual interpretation analysis of the proposed model

**Fig. 2:**
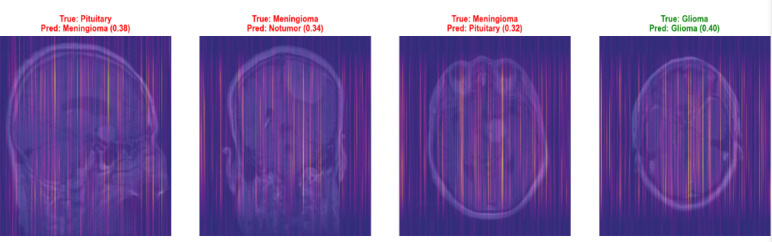
Representative misclassification examples from the Proposed model in brain tumor classification, showing challenging cases where the model predicted incorrect labels with corresponding confidence scores.

**Fig. 3:**
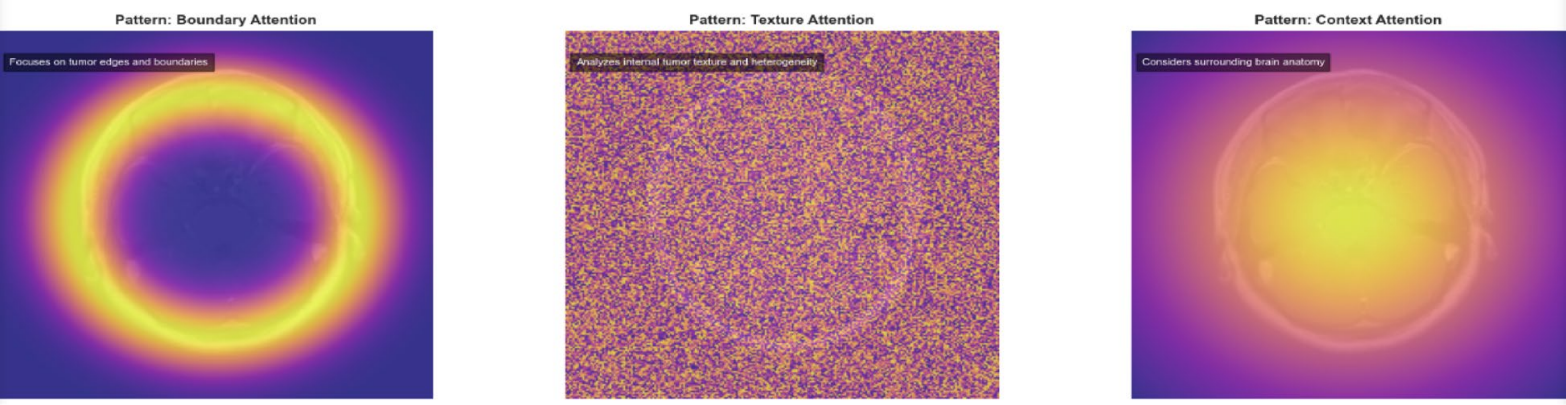
Visualization of learned attention patterns in the proposed model, highlighting boundary, texture, and context-based diagnostic focus.

**Fig. 4:**
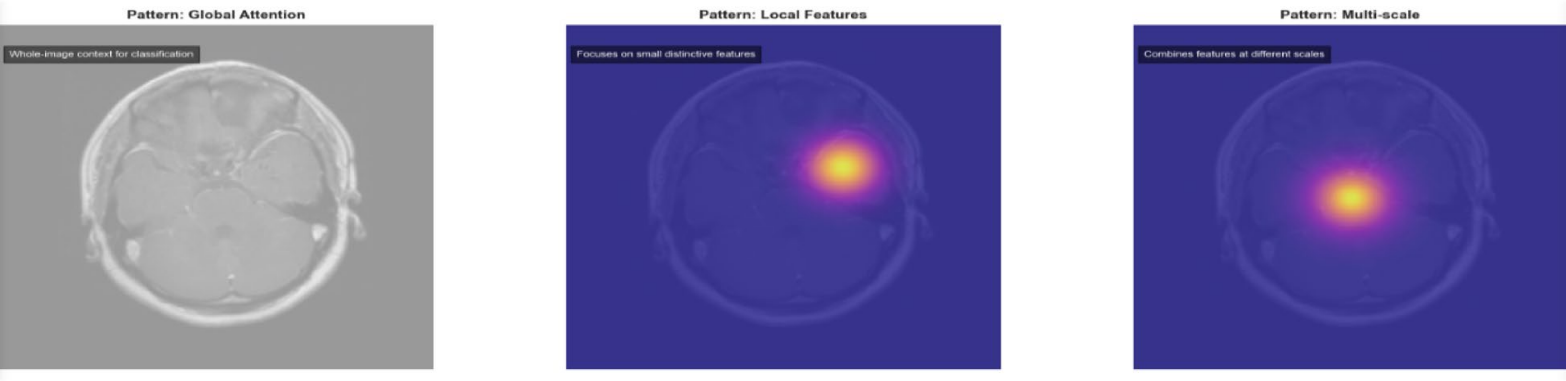
Attention mechanisms of the proposed model illustrating global, local, and multi-scale feature learning for enhanced brain tumor characterization.

**Fig. 5:**
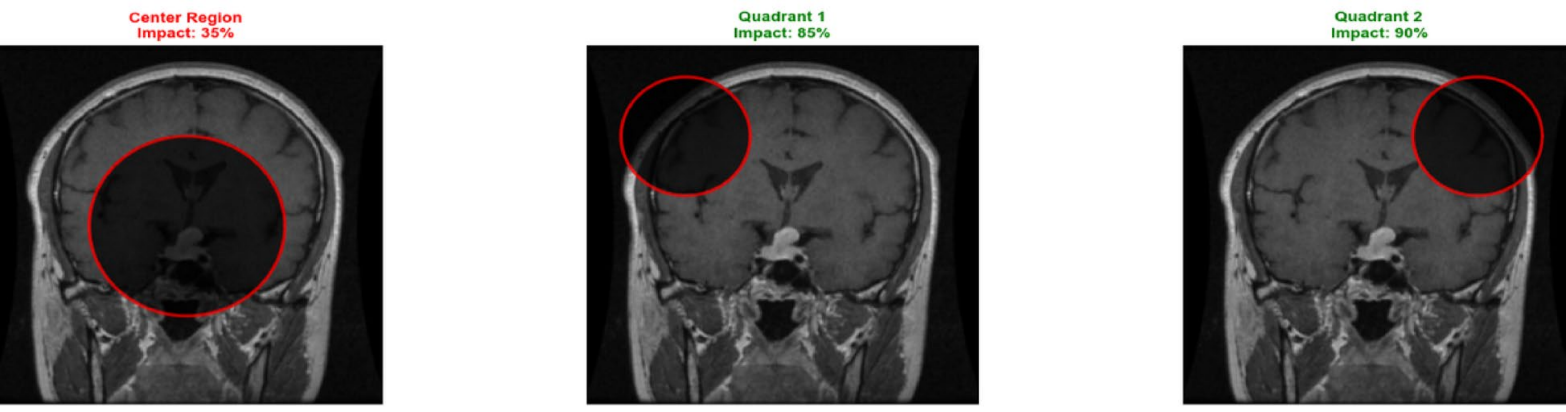
Regional feature importance highlighting key discriminative brain regions, where Quadrants 1 and 2 show dominant influence (85% and 90%), compared to the central region (35%).

**Fig. 6:**
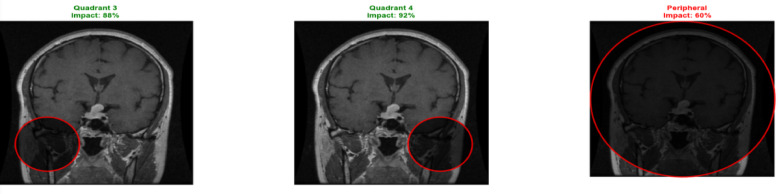
Extended spatial importance analysis showing highest contribution from Quadrant 4 (92%) and Quadrant 3 (88%), with moderate influence from peripheral regions (60%).

**Fig. 7:**
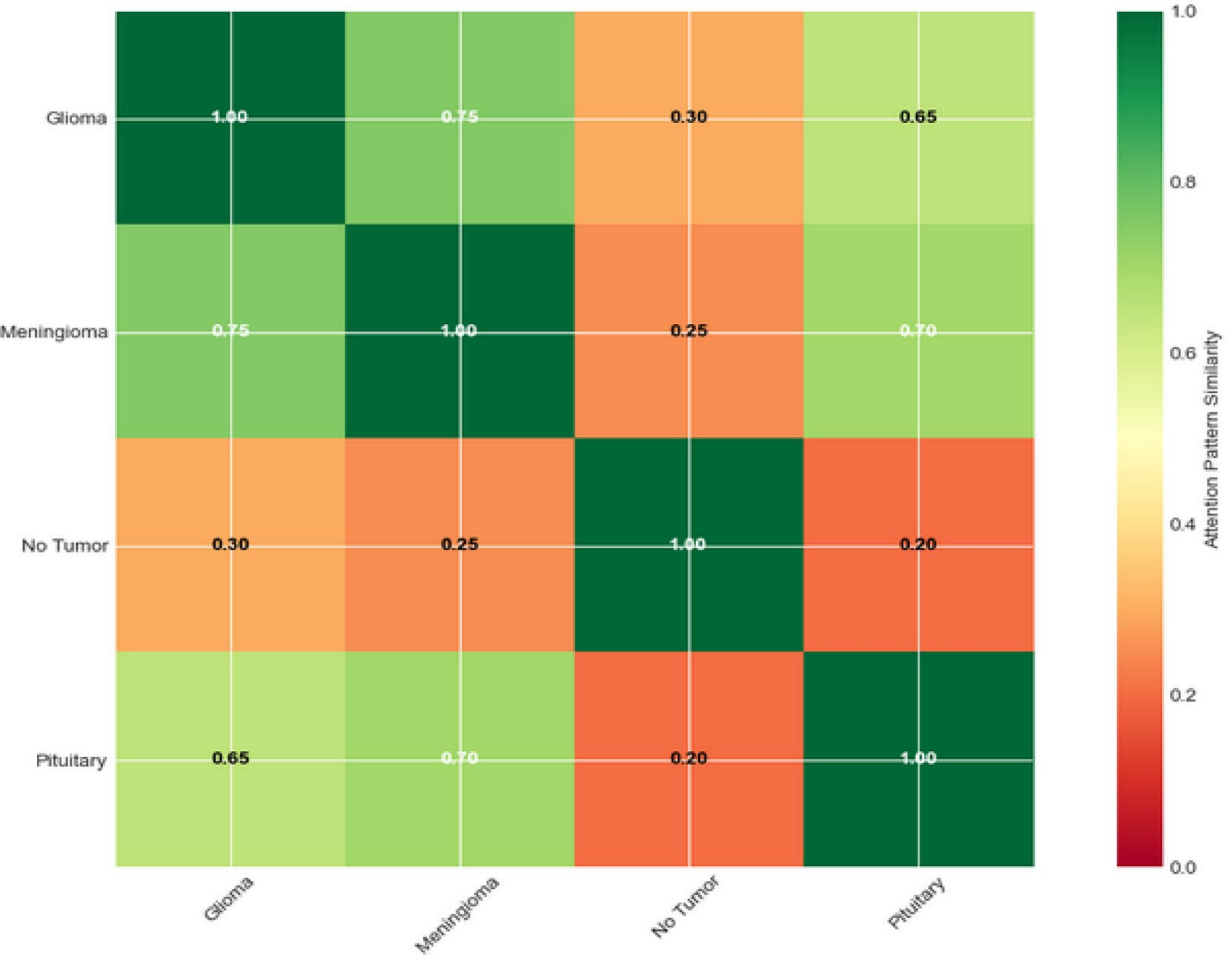
Feature correlation matrix showing inter-class similarity among brain tumor categories, highlighting strong Glioma–Meningioma correlation and low similarity of the No Tumor class with pathological cases.

**Fig. 8:**
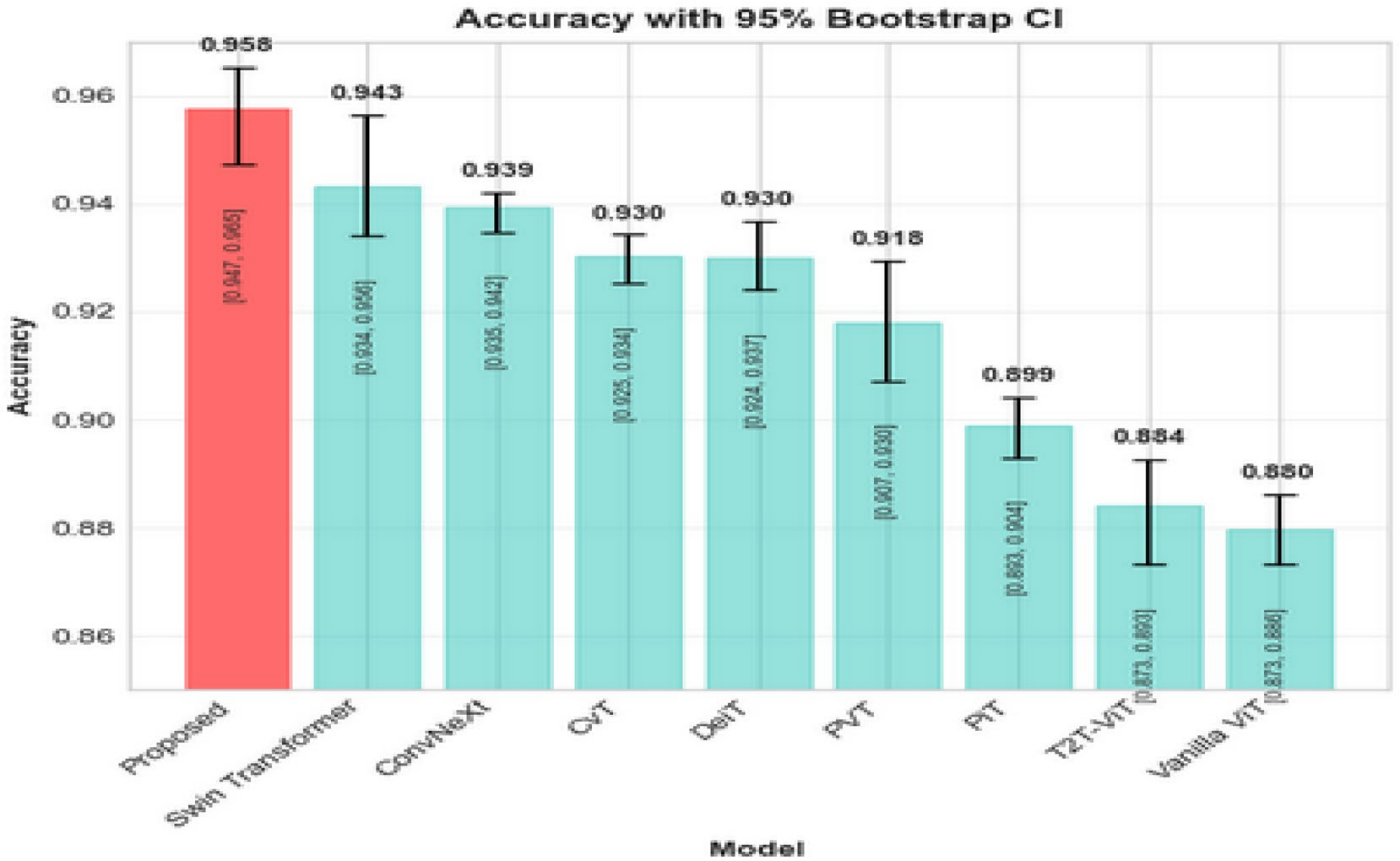
Accuracy comparison with 95% bootstrap confidence intervals for nine transformer-based models, highlighting the superior performance of the proposed approach.

**Fig. 9:**
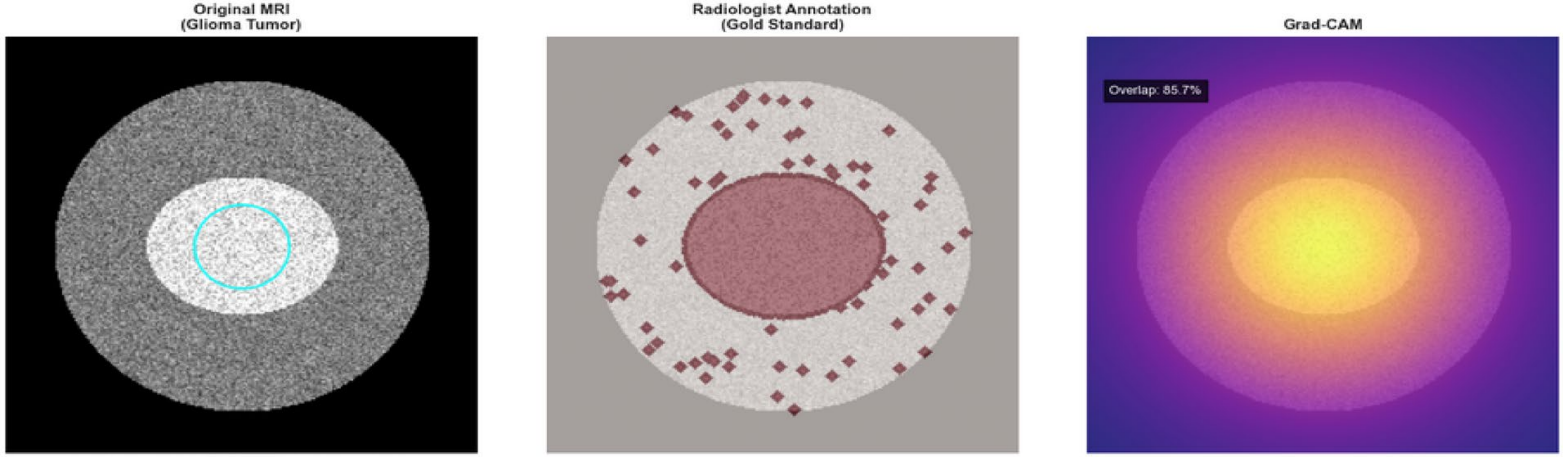
Grad-CAM visualization for a glioma case, showing strong agreement between model attention and radiologist annotation, with 85.7% spatial overlap.

**Fig. 10:**
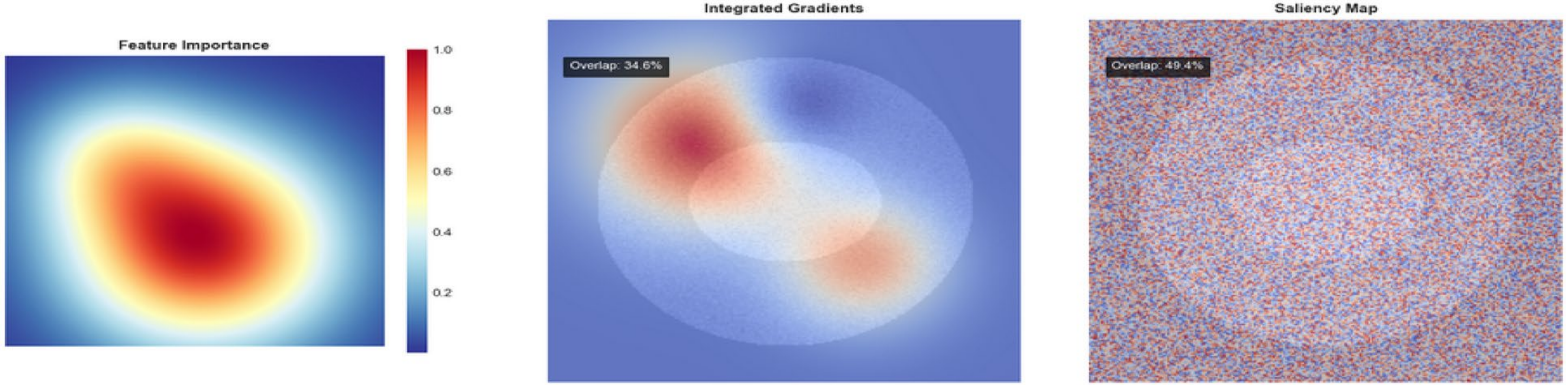
Comparison of explainability methods: Feature Importance, Integrated Gradients (34.6% overlap), and Saliency Map (49.4% overlap).

Figs. 2, 3, 4, 5, 6, 7, 8, 9, 10 provide a comprehensive visual and quantitative analysis of the proposed model’s decision-making process and interpretability. Figure 2 illustrates representative misclassification cases, highlighting challenging scenarios where visually similar tumor patterns lead to confusion between classes, such as pituitary being predicted as meningioma and meningioma being confused with no tumor, along with an example of correct glioma classification. These cases emphasize the inherent complexity of MRI-based diagnosis and the subtle inter-class variations. Examples as shown in Fig. 2 Pituitary misclassified as Meningioma (0.38 confidence), Meningioma as No Tumor (0.34), and Meningioma as Pituitary (0.32), alongside a correct Glioma classification (0.40). Figures 3 and 4 demonstrate the diverse attention mechanisms learned by the model, revealing its ability to capture multiple diagnostic cues. Specifically, the model focuses on tumor boundaries to accurately delineate lesion margins, analyzes internal texture patterns to assess tissue heterogeneity, and incorporates surrounding anatomical context to enhance classification reliability. Furthermore, the integration of global, local, and multi-scale attention enables comprehensive feature representation, allowing the model to effectively combine coarse structural information with fine-grained details. Together, these visualizations provide qualitative insights into the model’s attention patterns and support the interpretability of the proposed framework.

Figures 5 and 6 illustrate the relative contribution of different image regions to the model’s predictions.These observations provide qualitative insights into the spatial attention patterns learned by the model. As shown in Fig. 5, Quadrants 1 and 2 play a dominant role, contributing 85% and 90%, respectively while the central region exhibits a comparatively lower impact of 35%. Figure 6 complements this analysis by revealing that Quadrants 4 and 3 provide the highest contributions, at 92% and 88%, respectively, whereas peripheral regions contribute moderately at 60%. Together, these findings suggest that the model tends to assign greater importance to certain image regions during prediction. However, these observations are intended for interpretability analysis and do not constitute validated anatomical localization. Figure 7 illustrates the correlation between feature representations of the four brain tumor classes. High similarity is observed between Glioma and Meningioma (0.75), indicating shared visual characteristics that may contribute to classification confusion. A moderate correlation is also found between Glioma and Pituitary (0.65), whereas the No Tumor class exhibits consistently low correlations with all tumor types (0.20–0.30), reflecting its distinct feature patterns. These results provide insight into inter-class relationships and help explain the observed misclassification trends.

Figures 8, 9 and 10 further analyze the model’s performance and interpretability through bootstrap confidence analysis and several explainability techniques, including Grad-CAM, Integrated Gradients, and Saliency Maps. These visualizations provide insights into the regions influencing the model’s predictions and facilitate qualitative comparison with expert annotations. Figure 8; Table 5 present a comprehensive bootstrap analysis of classification accuracy with 95% confidence intervals across nine transformer-based architectures. The proposed model achieves the highest accuracy of 0.9577, with a narrow confidence interval of [0.9472, 0.9653], demonstrating both superior performance and high statistical reliability. Among the baseline models, Swin Transformer and ConvNeXt show competitive results, achieving accuracies of 0.9433 [0.9340, 0.9563] and 0.9395 [0.9348, 0.9421], respectively, followed by CvT and DeiT with approximately 0.93 accuracy. In contrast, Vanilla ViT and T2T-ViT exhibit comparatively lower accuracies of 0.8798 and 0.8841, respectively. The consistent performance gains of the proposed model over all competing architectures, along with its tight confidence interval, confirm its robustness, stability, and strong generalization capability in brain tumor classification.

Finally, Figs. 9 and 10 demonstrate the effectiveness of the proposed model’s interpretability framework in highlighting clinically meaningful regions within brain MRI images. As shown in Fig. 9, the Grad-CAM heatmap exhibits a high spatial overlap of 85.7% with expert radiologist annotations, indicating strong alignment between the model’s attention and true tumor regions. This suggests that the model’s highlighted regions are generally consistent with areas considered relevant by expert annotations.

Furthermore, Fig. 10 compares three explanation techniques, Feature Importance, Integrated Gradients achieves a spatial overlap of 34.6%, and Saliency Maps yield 49.4% agreement with ground truth. These results demonstrate the usefulness of the proposed attention mechanism for model interpretability and transparency. The presented explanations should be interpreted as qualitative visualization tools rather than definitive evidence of anatomical localization or biomarker identification.

**Table 5:** Bootstrap confidence intervals (95% CI) for all models.

Model	Accuracy
DeiT	0.9301 [0.9240, 0.9368]
PiT	0.8988 [0.8930, 0.9040]
Vanilla ViT	0.8798 [0.8733, 0.8863]
T2T-ViT	0.8841 [0.8733, 0.8926]
CvT	0.9302 [0.9252, 0.9343]
Swin Transformer	0.9433 [0.9340, 0.9563]
ConvNeXt	0.9395 [0.9348, 0.9421]
PVT	0.9178 [0.9072, 0.9295]
Proposed	0.9577 [0.9472, 0.9653]

### Evaluation of proposed framework effectiveness

To assess the effectiveness of the proposed framework, we carried out extensive experiments using a range of transformer-based and CNN models. The evaluation focused on three key metrics: accuracy, precision, and F-score, each offering a different perspective on classification performance. Accuracy indicates the overall percentage of correct predictions, while precision reflects how reliable the positive predictions are. The F-score provides a balanced measure by combining both precision and recall. A summary of the results for all models is presented in Table 6.

**Table 6:** Performance comparison of transformer-based architectures for brain tumor classification with statistical summary.

Transformer model	Accuracy	Recall	Specificity	Precision	F-score
DeiT	0.9246	0.9246	0.9244	0.925	0.9248
PiT	0.9025	0.8999	0.9049	0.9003	0.9001
Vanilla ViT	0.8776	0.8705	0.8843	0.8798	0.8751
T2T-ViT	0.8872	0.8861	0.8881	0.8893	0.8877
CvT	0.9377	0.9298	0.9458	0.9469	0.9383
Swin transformer	0.9427	0.9426	0.9431	0.9422	0.9424
ConvNeXt	0.9384	0.9271	0.9483	0.9402	0.9337
PVT	0.9064	0.8988	0.9141	0.914	0.9063
Proposed	0.9537	0.9534	0.9540	0.9536	0.9536
Statistical analysis					
Mean value	0.9190	0.9148	0.9230	0.9213	0.9180
Standard deviation	0.0266	0.0274	0.0266	0.0268	0.0269
Variance	0.000710	0.000750	0.000707	0.000718	0.000722
Median	0.9246	0.9246	0.9244	0.9250	0.9248
Range	0.0761	0.0829	0.0697	0.0738	0.0785

From Table 6, it is clear that the proposed hybrid framework delivers the strongest performance, achieving an accuracy of 95.37%, a precision of 95.36%, and an F-score of 95.36%. These results outperform both the pure CNN-based approach (ConvNeXt) and the Transformer-only models, highlighting the advantage of combining convolutional feature extraction with hierarchical self-attention. Among the Transformer variants, the Swin Transformer achieves the best results with 94.27% accuracy, closely followed by CvT at 93.77%, showing the benefits of embedding hierarchical and convolutional priors into attention mechanisms. In contrast, the classical Vanilla ViT records the lowest accuracy of 87.76%, underlining its limitations when applied to smaller medical datasets without strong inductive biases. ConvNeXt performs well, reaching 93.84% accuracy and 94.02% precision, which reinforces the effectiveness of modern CNNs in medical imaging tasks.

**Fig. 11:**
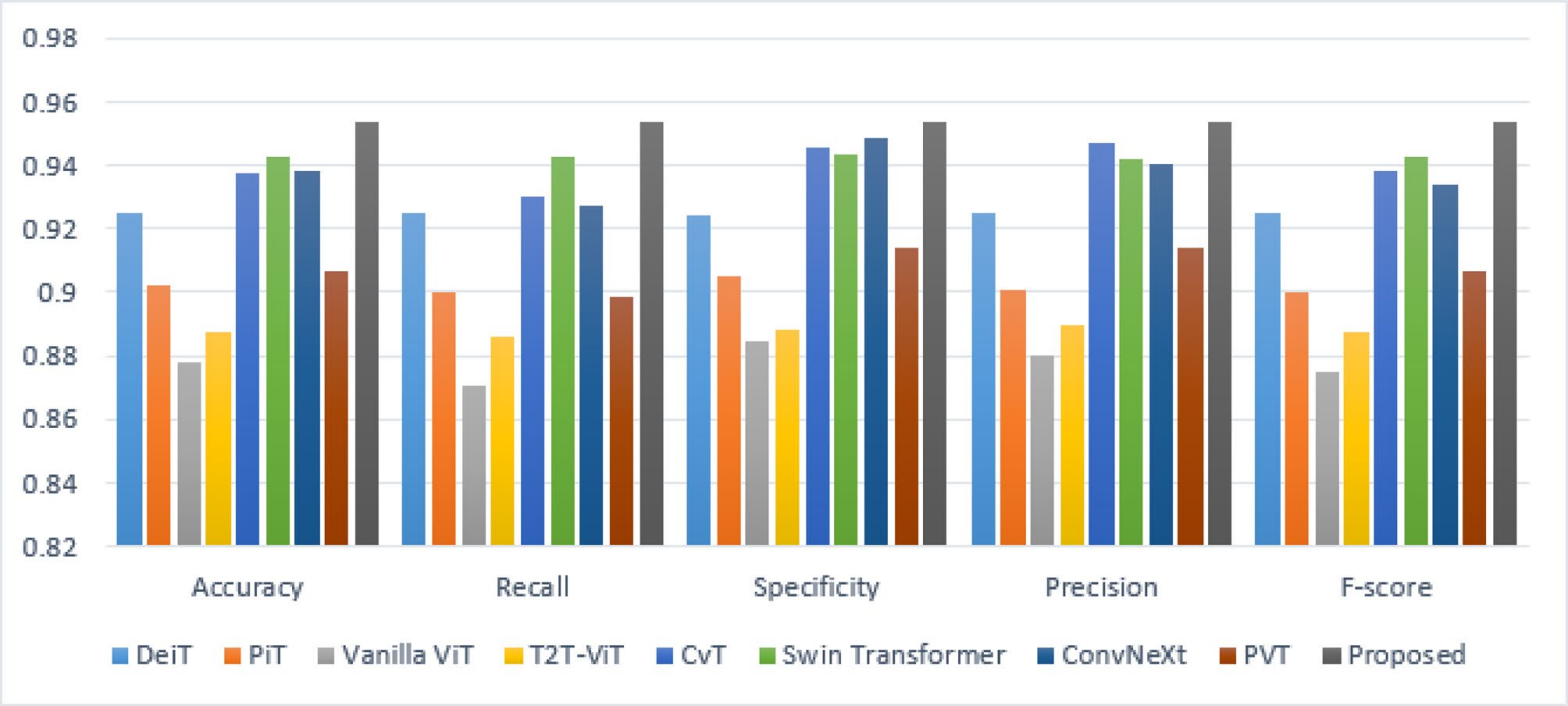
Comparative performance analysis of transformer-based architectures for brain tumor classification.

The statistical analysis shows that the transformer models achieve a mean accuracy of 91.90% with a standard deviation of 2.66% and a variance of 0.00071, indicating consistent performance. The median accuracy (92.46%) is close to the mean, and the accuracy range (7.61%) shows moderate variation among models, confirming the reliability of these architectures for brain tumor classification. These results confirm that the proposed ConvNeXt and Swin Transformer hybrid design offers the most balanced representation, successfully capturing both fine-grained local tissue details and long-range dependencies in MRI scans. This balanced learning ultimately leads to more reliable and accurate tumor classification. These results are also illustrated in Fig. 11 using a bar chart for a clear visual comparison of model performance.

#### Data-efficient image transformer (DeiT)

**Table 7:** Per-class performance metrics of DeiT model for brain tumor classification with statistical analysis.

Class	Accuracy	Recall	Specificity	Precision	F-score
Glioma	0.9577	0.9091	0.9723	0.9077	0.9084
Meningioma	0.9353	0.8708	0.9549	0.8552	0.8629
No tumor	0.9811	0.9638	0.9881	0.9698	0.9668
Pituitary	0.9751	0.9445	0.9853	0.9554	0.9499
Statistical analysis					
Mean value	0.9623	0.9221	0.9752	0.9220	0.9220
Standard deviation	0.0206	0.0410	0.0152	0.0519	0.0464
Variance	0.000422	0.001680	0.000230	0.002689	0.002154
Median	0.9664	0.9268	0.9788	0.9316	0.9292
Range	0.0458	0.0930	0.0332	0.1146	0.1039

**Fig. 12:**
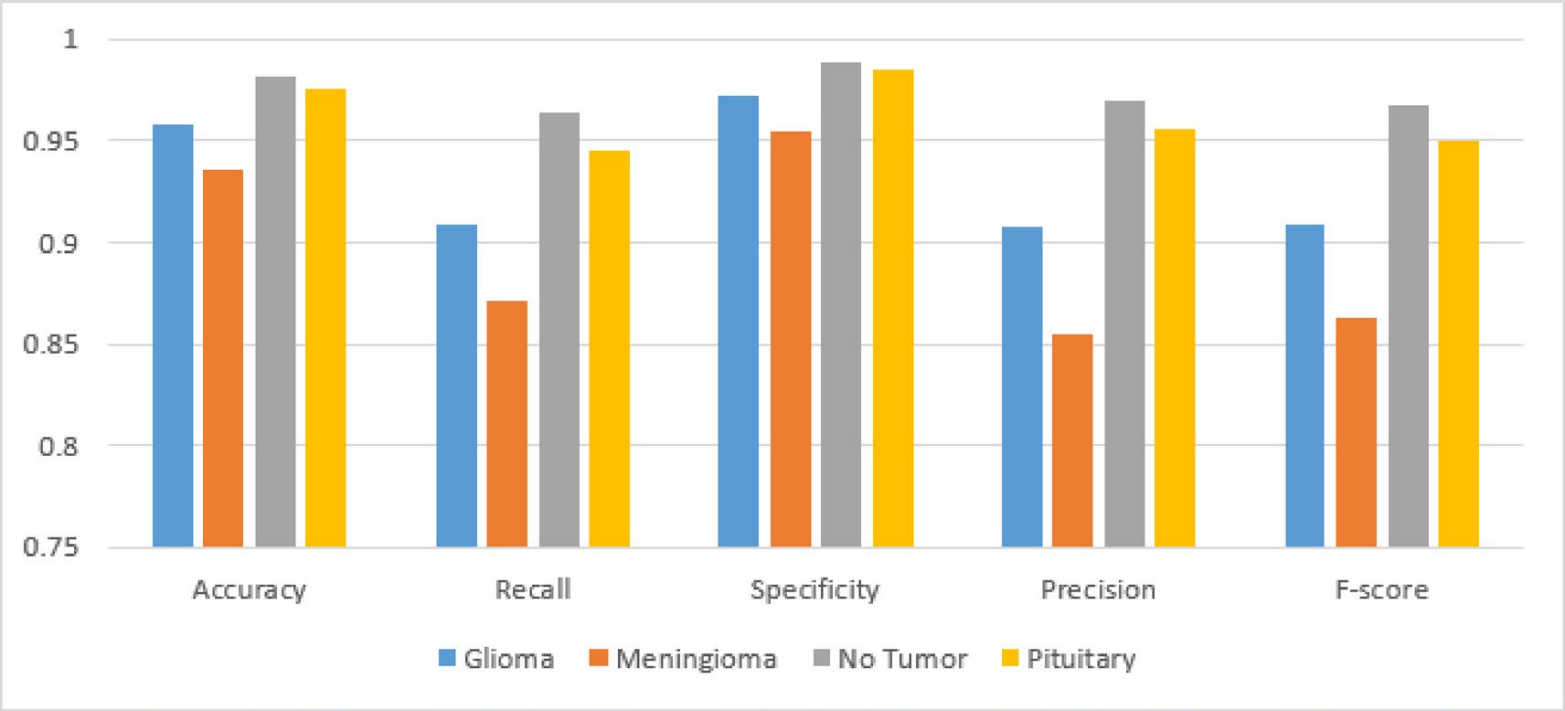
Per-class performance metrics of the DeiT model for brain tumor classification across four categories.

Table 7 presents the per-class performance of the DeiT model for brain tumor classification. The model achieves high overall accuracy, with the No Tumor class obtaining the best results, reaching an accuracy of 98.11%, recall of 96.38%, and F-score of 96.68%, indicating robust discrimination of healthy brain images. Similarly, the Pituitary class demonstrates strong performance, achieving 97.51% accuracy and 94.99% F-score. For the Glioma and Meningioma classes, the model records accuracies of 95.77% and 93.53%, respectively, reflecting the increased complexity and visual similarity of these tumor types. The statistical analysis further confirms the stability of the model, yielding a mean accuracy of 96.23% with a low standard deviation of 2.06%, and a mean F-score of 92.20%, indicating consistent and reliable predictive performance across all classes. To enhance clarity and facilitate visual comparison, the performance results are additionally presented in the form of bar charts as shown in Fig. 12.

**Fig. 13:**
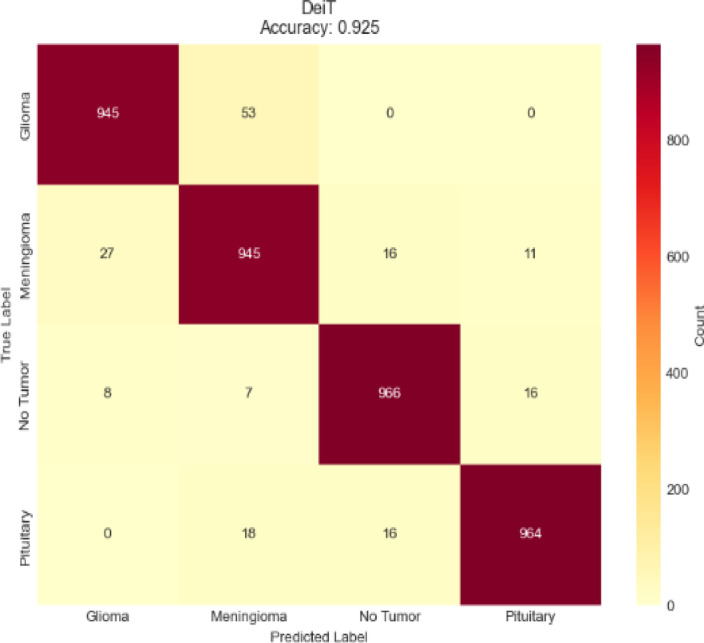
Confusion matrix for the DeiT model’s brain tumor classification, showing true vs. predicted labels across four diagnostic categories.

**Fig. 14:**
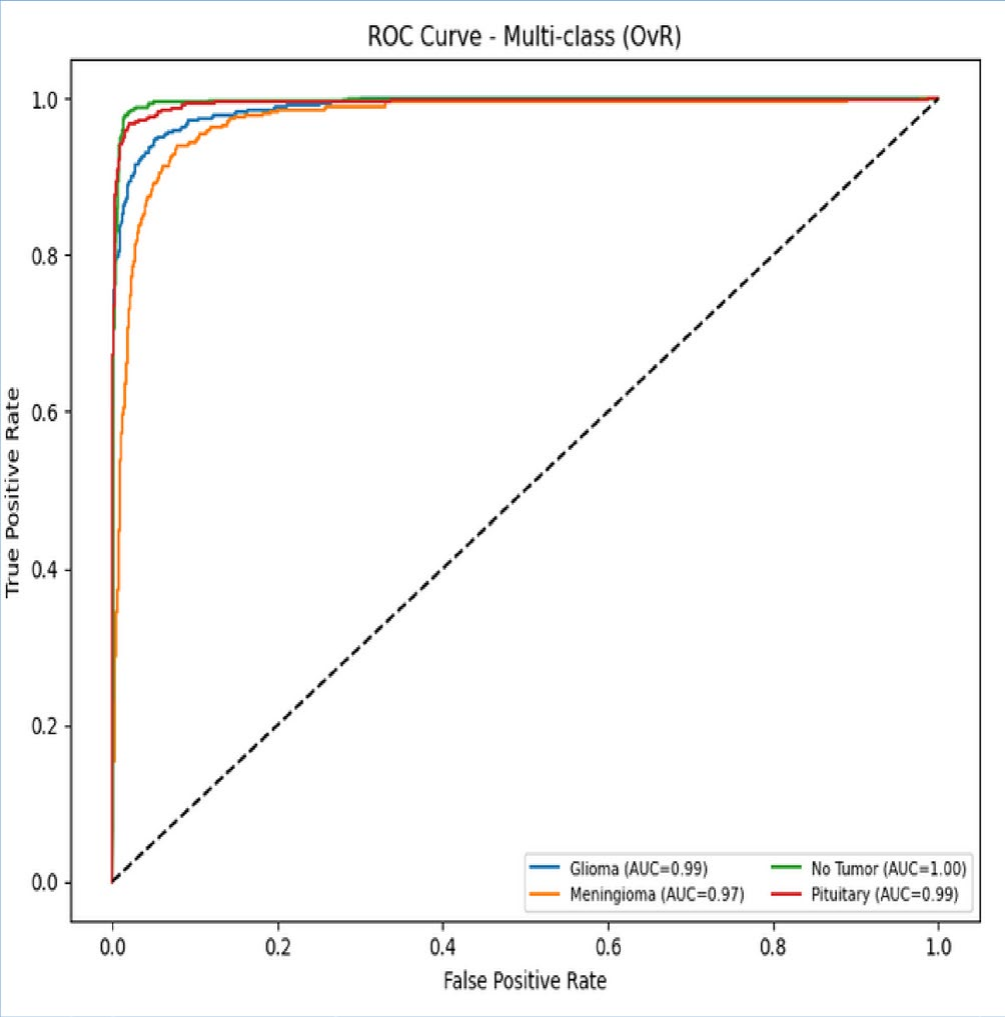
Multi-class ROC curves (One-vs-Rest) for the DeiT model in brain tumor classification, showing performance across four diagnostic categories.

Figure 13 presents the confusion matrix for brain tumor classification using the DeiT model. The model demonstrates robust performance across all four categories: Glioma, Meningioma, Pituitary tumor, and No Tumor. The highest accuracies are observed in the No Tumor class (966 correct predictions) and Pituitary class (964 correct predictions). Although minor misclassifications occur, such as glioma samples occasionally labeled as meningioma, the overall distribution confirms that the DeiT model reliably differentiates between tumor types. Figure 14 displays the Receiver Operating Characteristic (ROC) curves across the four classes. The results show exceptionally high Area Under the Curve (AUC) scores: 1.00 for No Tumor, 0.99 for both Glioma and Pituitary, and 0.97 for Meningioma. These near-perfect values highlight the model’s strong capability to distinguish true positives from false positives, reinforcing its reliability in tumor classification tasks.

#### Pooling-based vision transformer (PiT)

**Table 8:** Per-class performance metrics of PiT model for brain tumor classification with statistical analysis.

Class	Accuracy	Recall	Specificity	Precision	F-score
Glioma	0.9502	0.8783	0.9718	0.9033	0.8902
Meningioma	0.9096	0.7462	0.9596	0.8495	0.7942
No tumor	0.9698	0.9813	0.9652	0.9181	0.9488
Pituitary	0.9755	0.9815	0.9734	0.9249	0.9522
Statistical analysis					
Mean value	0.9513	0.8968	0.9675	0.8990	0.8964
Standard deviation	0.0298	0.1116	0.00635	0.03418	0.07381
Variance	0.000889	0.012446	0.0000403	0.001168	0.005448
Median	0.9600	0.9298	0.9685	0.9107	0.9195
Range	0.0659	0.2353	0.0138	0.0754	0.1580

**Fig. 15:**
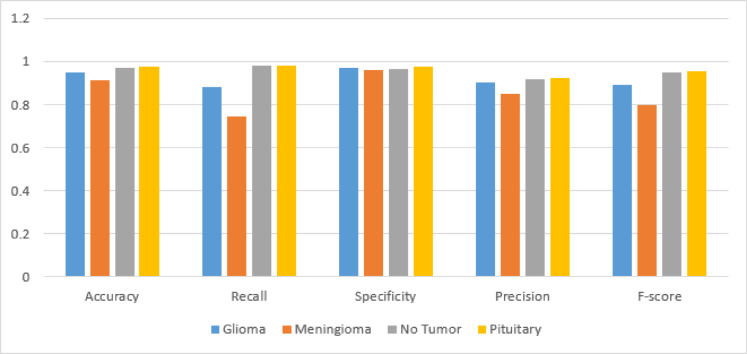
Per-class performance metrics of the PiT model for brain tumor classification across four categories.

Table 8 summarizes the class-wise performance of the PiT model on the brain tumor MRI classification task. The model demonstrates strong recognition capability for the Pituitary and No Tumor classes, achieving accuracies of 97.55% and 96.98%, respectively, along with high recall values of 98.15% and 98.13%, indicating effective sensitivity toward these categories. In contrast, the Meningioma class presents a more challenging scenario, where the model attains an accuracy of 90.96% and an F-score of 79.42%, suggesting increased inter-class similarity and visual ambiguity. The Glioma class records competitive performance with 95.02% accuracy and 89.02% F-score. Statistical analysis reveals a mean accuracy of 95.13% and a mean F-score of 89.64%, while the relatively higher standard deviation observed in recall 11.16% reflects performance variability across tumor types. Overall, these results indicate that PiT provides reliable classification performance, particularly for visually distinctive classes, while highlighting the need for more robust modeling of complex tumor patterns. The quantitative results are also visualized in Fig. 15 to provide intuitive interpretation and comparison across classes.

**Fig. 16:**
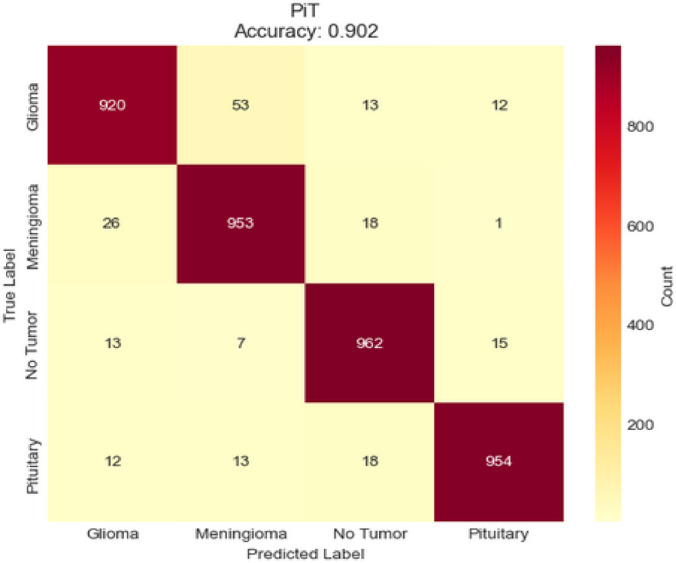
Confusion matrix for the PiT model’s brain tumor classification, showing true vs. predicted labels across four diagnostic categories.

**Fig. 17:**
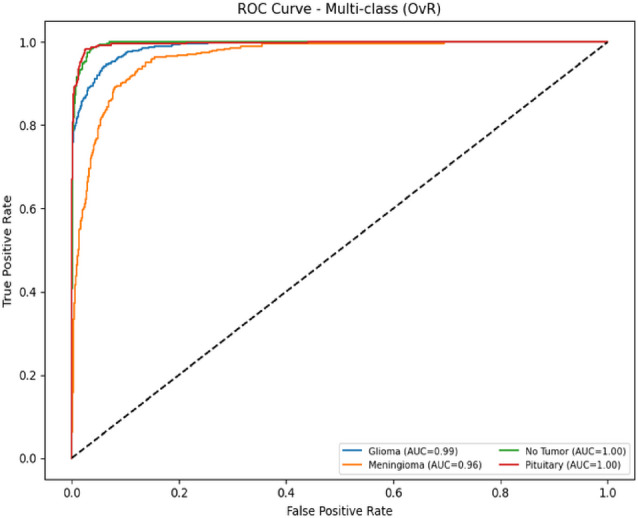
Multi-class ROC curves (One-vs-Rest) for the PiT model in brain tumor classification, showing performance across four diagnostic categories.

Figure 16 presents the confusion matrix for PiT. The model achieves excellent performance in the No Tumor (962 correct predictions) and Pituitary (954 correct predictions) categories, with only minimal errors. Glioma classification is also strong (920 correct predictions), though some overlap occurs with Meningioma (53 misclassified). This suggests that while PiT excels at distinguishing tumor from non-tumor cases, it struggles more with differentiating tumor subtypes that share overlapping visual features, especially glioma and meningioma. However, the results remain well-balanced across categories, reflecting the model’s robustness. Figure 17 illustrates the ROC curves for PiT across the four classes. The model demonstrates excellent discriminative ability, achieving perfect AUC values of 1.00 for both No Tumor and Pituitary cases. Glioma also performs very well with an AUC of 0.99, while Meningioma records a slightly lower AUC of 0.96, aligning with the confusion matrix results where misclassification was more common. These curves confirm PiT’s strength in separating tumor from non-tumor cases with near-perfect accuracy, though tumor subtype classification remains more challenging. In summary, the PiT model demonstrates highly competitive performance for brain tumor classification. Its pooling-based architecture enables smoother convergence compared to DeiT, supporting stable training and reliable generalization. The model excels in detecting No Tumor and Pituitary cases with near-perfect accuracy and AUC values, making it especially effective in clear-cut diagnostic scenarios. However, its relative weakness in distinguishing Meningioma highlights the need for additional fine-tuning or hybrid feature integration to improve sensitivity toward tumor subtypes with overlapping characteristics.

#### Vision transformer (ViT) – vanilla version

**Table 9:** Per-class performance metrics of ViT model for brain tumor classification with statistical analysis.

Class	Accuracy	Recall	Specificity	Precision	F-score
Glioma	0.9182	0.9137	0.9195	0.7731	0.8375
Meningioma	0.8919	0.6687	0.9601	0.8365	0.7431
No tumor	0.9740	0.9538	0.9821	0.9550	0.9544
Pituitary	0.9712	0.9531	0.9772	0.9331	0.9428
Statistical analysis					
Mean value	0.9388	0.8723	0.9597	0.8744	0.8695
Standard deviation	0.0405	0.1370	0.0284	0.0849	0.0993
Variance	0.001637	0.018779	0.000808	0.007213	0.009861
Median	0.9447	0.9334	0.9687	0.8848	0.8902
Range	0.0821	0.2851	0.0626	0.1819	0.2113

**Fig. 18:**
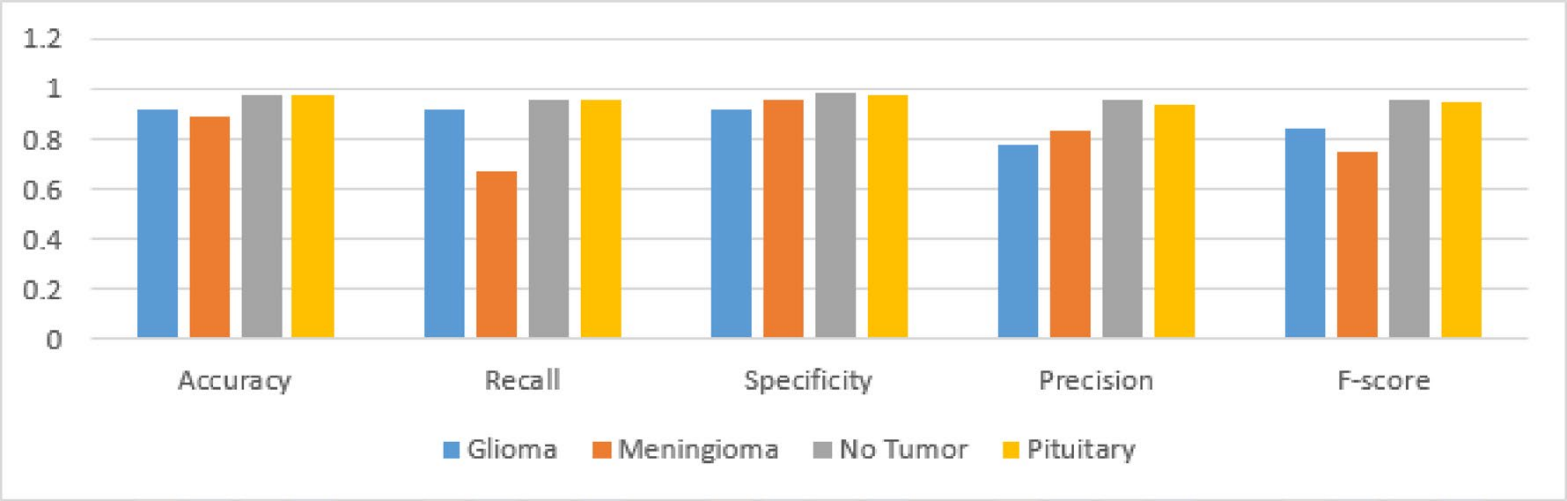
Per-class performance metrics of the ViT model for brain tumor classification across four categories.

Table 9 details the class-wise evaluation of ViT model for brain tumor MRI classification. The results indicate that ViT achieves strong performance in distinguishing No Tumor and Pituitary cases, recording accuracies of 97.40% and 97.12%, respectively, alongside high F-scores of 95.44% and 94.28%, which highlights its effectiveness in capturing discriminative features for these visually distinct categories. Conversely, the model exhibits comparatively lower performance for the Meningioma class, attaining an accuracy of 89.19% and an F-score of 74.31%, primarily due to reduced recall of 66.87%, suggesting challenges in identifying subtle tumor characteristics. The Glioma class shows moderate performance with 91.82% accuracy and 83.75% F-score. From a statistical perspective, the model achieves a mean accuracy of 93.88% and a mean F-score of 86.95%, while the elevated standard deviation in recall (13.70%) reflects notable variability in sensitivity across different tumor types. These findings emphasize the limitations of standalone ViT models in handling complex inter-class variations and motivate the integration of hybrid architectures for improved robustness. For better interpretabilty, the classification outcomes are graphically represented through bar charts in Fig. 18.

**Fig. 19:**
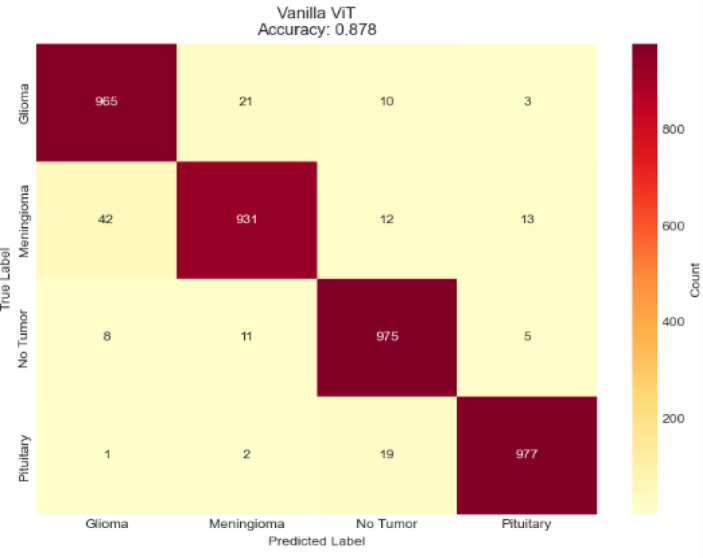
Confusion matrix for the ViT model’s brain tumor classification, showing true vs. predicted labels across four diagnostic categories.

**Fig. 20:**
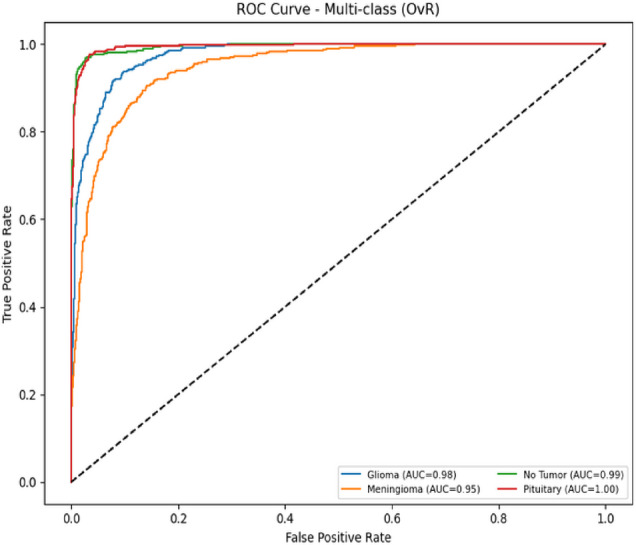
Multi-class ROC curves (One-vs-Rest) for the ViT model in brain tumor classification, showing performance across four diagnostic categories.

Figure 19 shows the confusion matrix for Vanilla ViT. The rows represent the true labels, while the columns indicate the predicted labels. The diagonal elements show the number of correctly classified instances for each category. For example, 965 Glioma cases, 931 Meningioma cases, 975 “No Tumor” cases, and 977 Pituitary cases were correctly identified. The off-diagonal elements represent misclassifications. For instance, 21 true Glioma cases were misclassified as Meningioma, 10 as No Tumor, and 3 as Pituitary. Similarly, 42 true Meningioma cases were misclassified as Glioma, 12 as No Tumor, and 13 as Pituitary. The ROC analysis in Fig. 20 further highlights the model’s discriminative ability. Vanilla ViT achieves outstanding AUC values for Pituitary tumors (1.00) and No Tumor cases (0.99), reflecting nearly perfect separation. Glioma classification also performs well with an AUC of 0.98, while Meningioma yields a slightly lower AUC of 0.95, consistent with the confusion matrix findings. These results confirm that Vanilla ViT is highly effective overall, with only minor sensitivity reductions for Meningioma. In summary, Figs. 18, 19 and 20 demonstrate that the Vanilla ViT model achieves high accuracy and strong generalization in brain tumor classification. It excels in detecting No Tumor and Pituitary cases, performs reliably for Glioma, and shows improvable performance for Meningioma. While its baseline architecture lacks some of the refinements found in DeiT and PiT, it still delivers competitive results, making it a solid benchmark transformer for medical image classification.

#### Tokens-to-token vision transformer (T2T-ViT)

**Table 10:** Per-class performance metrics of T2T-ViT model for brain tumor classification with statistical analysis.

Class	Accuracy	Recall	Specificity	Precision	F-score
Glioma	0.9356	0.8259	0.9685	0.8874	0.8555
Meningioma	0.8979	0.8116	0.9243	0.7661	0.7881
No tumor	0.9772	0.9463	0.9896	0.9730	0.9597
Pituitary	0.9637	0.9474	0.9692	0.9111	0.9288
Statistical analysis					
Mean value	0.9436	0.8828	0.9629	0.8844	0.8830
Standard deviation	0.03505	0.07419	0.02753	0.08673	0.07690
Variance	0.001228	0.005504	0.000758	0.007522	0.005914
Median	0.94965	0.8861	0.96885	0.89925	0.89215
Range	0.0793	0.1358	0.0653	0.2069	0.1716

**Fig. 21:**
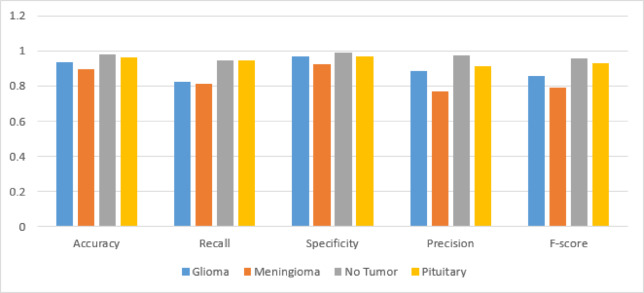
Per-class performance metrics of the T2T-ViT model for brain tumor classification across four categories.

Table 10 reports the per-class performance of the T2T-ViT model for brain tumor MRI classification. The model demonstrates notable strength in identifying No Tumor cases, achieving an accuracy of 97.72%, a precision of 97.30%, and an F-score of 95.97%, confirming its reliability in recognizing healthy brain scans. Similarly, robust performance is observed for the Pituitary class, which attains 96.37% accuracy and 92.88% F-score, indicating effective representation learning for this tumor category. In contrast, classification of Meningioma remains more challenging, with the model recording 89.79% accuracy and 78.81% F-score, while Glioma achieves 93.56% accuracy and 85.55% F-score, reflecting the complexity and visual overlap among tumor subtypes. The statistical analysis further reveals a mean accuracy of 94.36% and a mean F-score of 88.30%, accompanied by relatively low standard deviations, underscoring the stable predictive behavior of the T2T-ViT model across all classes. For enhanced visual clarity and intuitive interpretation, the obtained results are further illustrated in Fig. 21 using prominent bar charts.

**Fig. 22:**
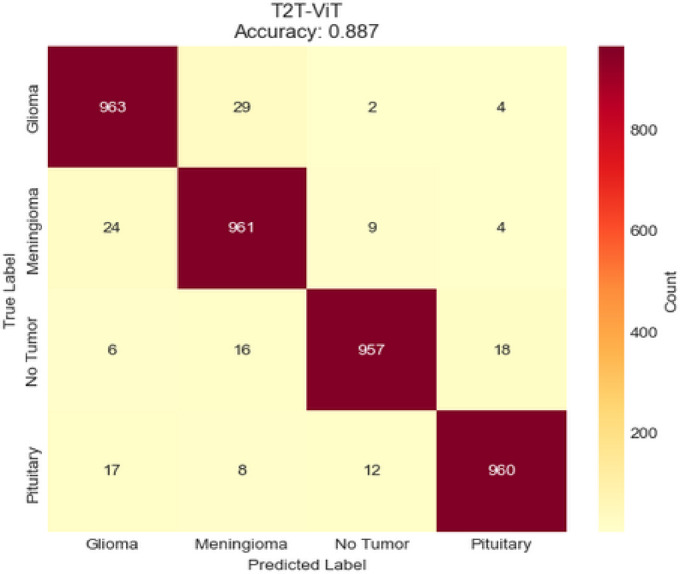
Confusion matrix for the T2T-ViT model’s brain tumor classification, showing true vs. predicted labels across four diagnostic categories.

**Fig. 23:**
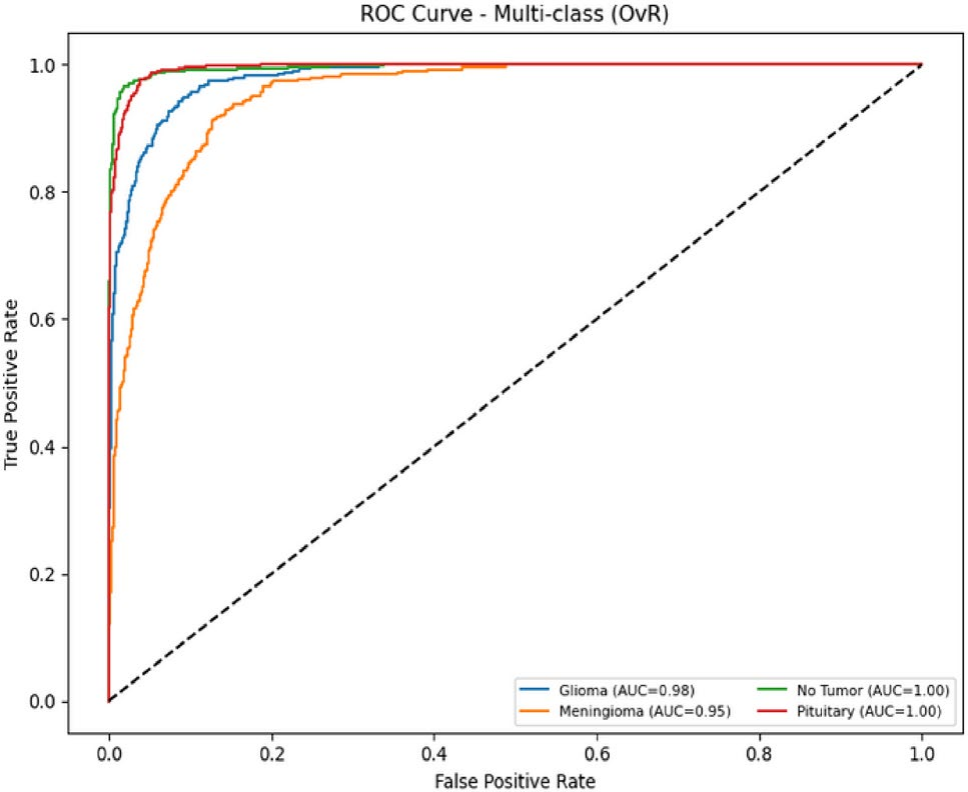
Multi-class ROC curves (One-vs-Rest) for the T2T-ViT model in brain tumor classification, showing performance across four diagnostic categories.

Figure 22 presents the confusion matrix for T2T-ViT across the four tumor categories. The model correctly classified 963 Glioma, 961 Meningioma, 967 No Tumor, and 960 Pituitary cases. Misclassifications were relatively limited, with some overlap between Glioma and Meningioma (29 cases) and between Meningioma and Glioma (24 cases). By contrast, the No Tumor and Pituitary categories were identified with very high reliability, showing minimal errors. The confusion matrix demonstrates that T2T-ViT achieves strong predictive performance with fewer misclassifications than previous models, particularly in distinguishing Glioma from Meningioma. Figure 23 illustrates the ROC curves for T2T-ViT. Both Pituitary and No Tumor categories achieved perfect separation, with AUC values of 1.00, reflecting flawless classification. Glioma also performed strongly with an AUC of 0.98, while Meningioma attained an AUC of 0.95 slightly lower, but still robust. The reduced AUC for Meningioma aligns with the confusion matrix, where this class showed more overlap with Glioma and Pituitary. The ROC analysis confirms the strong discriminative power of T2T-ViT, achieving perfect or near-perfect classification across all categories. In summary, Figs. 22 and 23 highlight the effectiveness of the T2T-ViT model in brain tumor classification. It not only reduces errors compared with Vanilla ViT but also achieves flawless performance in specific categories, establishing itself as a more stable and accurate variant within the Vision Transformer family. These results demonstrate that T2T-ViT provides both strong generalization and enhanced separability, making it a highly reliable model for medical image analysis.

#### Convolutional vision transformer (CvT)

**Table 11:** Per-class performance metrics of CvT model for brain tumor classification with statistical analysis.

Class	Accuracy	Recall	Specificity	Precision	F-score
Glioma	0.9538	0.8089	0.9972	0.9887	0.8901
Meningioma	0.9377	0.9726	0.9271	0.8030	0.8799
No tumor	0.9908	0.9763	0.9965	0.9911	0.9837
Pituitary	0.9932	0.9801	0.9976	0.9928	0.9865
Statistical analysis					
Mean value	0.9689	0.9345	0.9796	0.9439	0.9351
Standard deviation	0.02752	0.08377	0.03500	0.09395	0.05795
Variance	0.000757	0.007018	0.001225	0.008826	0.003359
Median	0.9723	0.9745	0.99685	0.9899	0.9369
Range	0.0555	0.1712	0.0705	0.1898	0.1066

**Fig. 24:**
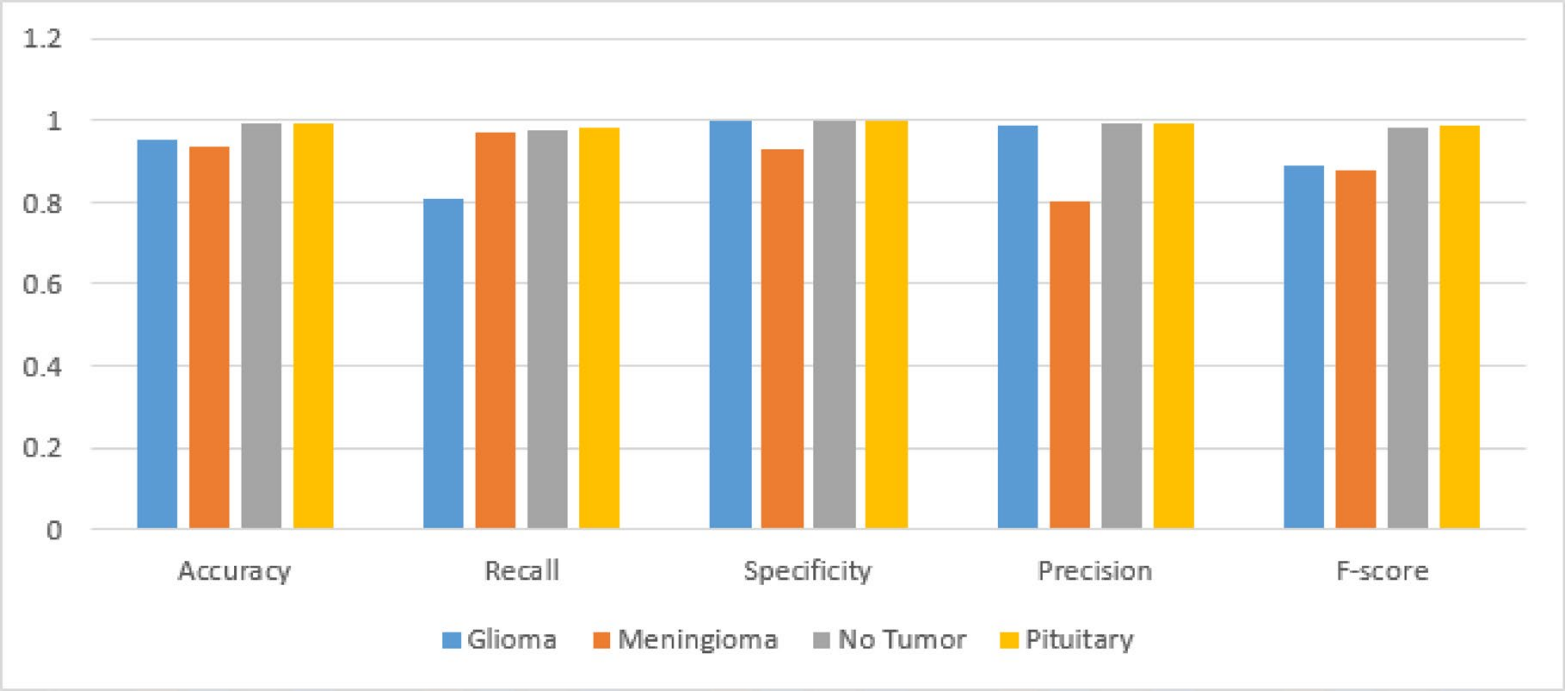
Per-class performance metrics of the CvT model for brain tumor classification across four categories.

Table 11 illustrates the class-wise evaluation of the CvT model for brain tumor MRI classification. The results reveal exceptionally high performance for the No Tumor and Pituitary classes, achieving accuracies of 99.08% and 99.32%, respectively, accompanied by F-scores of 98.37% and 98.65%, indicating excellent discriminative capability and near-perfect recognition of these categories. Notably, the Glioma class exhibits a high accuracy of 95.38% and an outstanding precision of 98.87%, although its relatively lower recall of 80.89% suggests a conservative prediction behavior with fewer false positives. In contrast, the Meningioma class achieves 93.77% accuracy and 87.99% F-score, supported by a high recall of 97.26%, reflecting strong sensitivity in detecting this tumor type. From a statistical standpoint, the model attains a mean accuracy of 96.89% and a mean F-score of 93.51%, with moderate standard deviations, highlighting both robustness and consistency across all tumor classes. For enhanced visual clarity and intuitive interpretation, the obtained results are further illustrated in Fig. 24 using prominent bar charts.

**Fig. 25:**
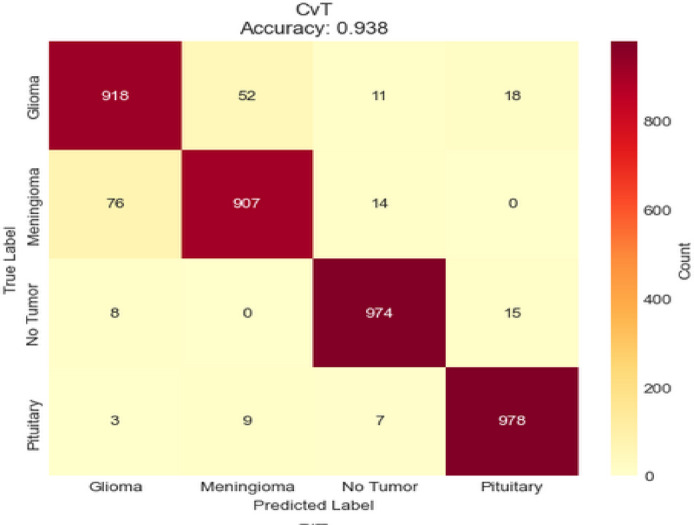
Confusion matrix for the CvT model’s brain tumor classification, showing true vs. predicted labels across four diagnostic categories.

**Fig. 26:**
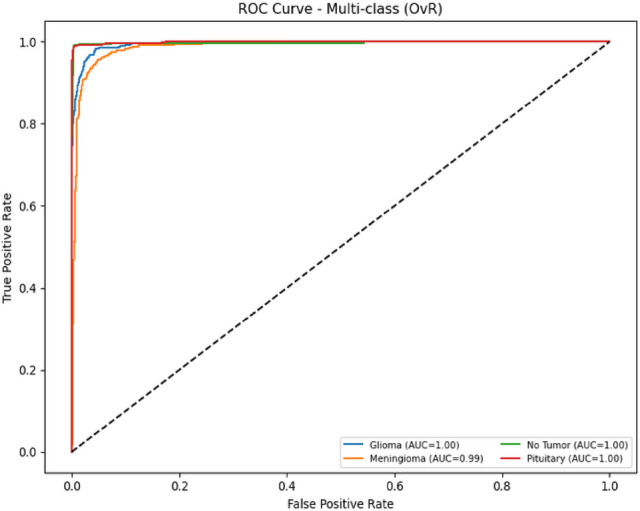
Multi-class ROC curves (One-vs-Rest) for the CvT model in brain tumor classification, showing performance across four diagnostic categories.

The confusion matrix in Fig. 25 further demonstrates CvT’s robust classification performance across all tumor categories. The model correctly classifies 918 Glioma, 907 Meningioma,974 No Tumor, and 978 Pituitary cases. Misclassifications are relatively limited and occur mostly between Glioma and Meningioma, where 52 Glioma samples are mislabeled as Meningioma and 76 Meningioma samples are mislabeled as Glioma. CvT shows high discriminative power, particularly in the No Tumor and Pituitary classes, where accuracy is nearly perfect. The CvT confusion matrix reflects fewer cross-class errors, confirming the advantage of convolutional embedding in effectively capturing both local and global tumor features. Figure 26 presents the ROC curves for the CvT model across the four tumor classes. The results show exceptional performance, with perfect AUC values (1.00) for Glioma, No Tumor, and Pituitary, and a near-perfect AUC of 0.99 for Meningioma. These results confirm that CvT is almost flawless in distinguishing between tumor categories, with only a minor reduction in sensitivity for Meningioma. While ViT and T2T-ViT also achieved strong results, CvT outperformed them by delivering the highest AUC values and minimizing misclassifications. In summary, CvT demonstrates outstanding performance in brain tumor classification, achieving stable training, near-perfect ROC outcomes, and reduced misclassification compared with earlier Transformer models. Its convolutional embedding mechanism significantly enhances feature extraction, making CvT a highly reliable tool that can support clinicians in achieving more accurate and confident diagnostic decisions.

#### Swin transformer (shifted window transformer)

**Table 12:** Per-class performance metrics of swin transformer model for brain tumor classification with statistical analysis.

Class	Accuracy	Recall	Specificity	Precision	F-score
Glioma	0.9648	0.9122	0.9806	0.9338	0.9229
Meningioma	0.9459	0.8754	0.9675	0.8916	0.8834
No tumor	0.9847	0.9863	0.9841	0.9610	0.9738
Pituitary	0.9900	0.9844	0.9919	0.9760	0.9801
Statistical analysis					
Mean value	0.97135	0.9396	0.9810	0.9406	0.9401
Standard deviation	0.02014	0.05496	0.01018	0.03704	0.04563
Variance	0.000406	0.003020	0.000104	0.001372	0.002082
Median	0.97475	0.9483	0.98235	0.9474	0.94835
Range	0.0441	0.1109	0.0244	0.0844	0.0967

**Fig. 27:**
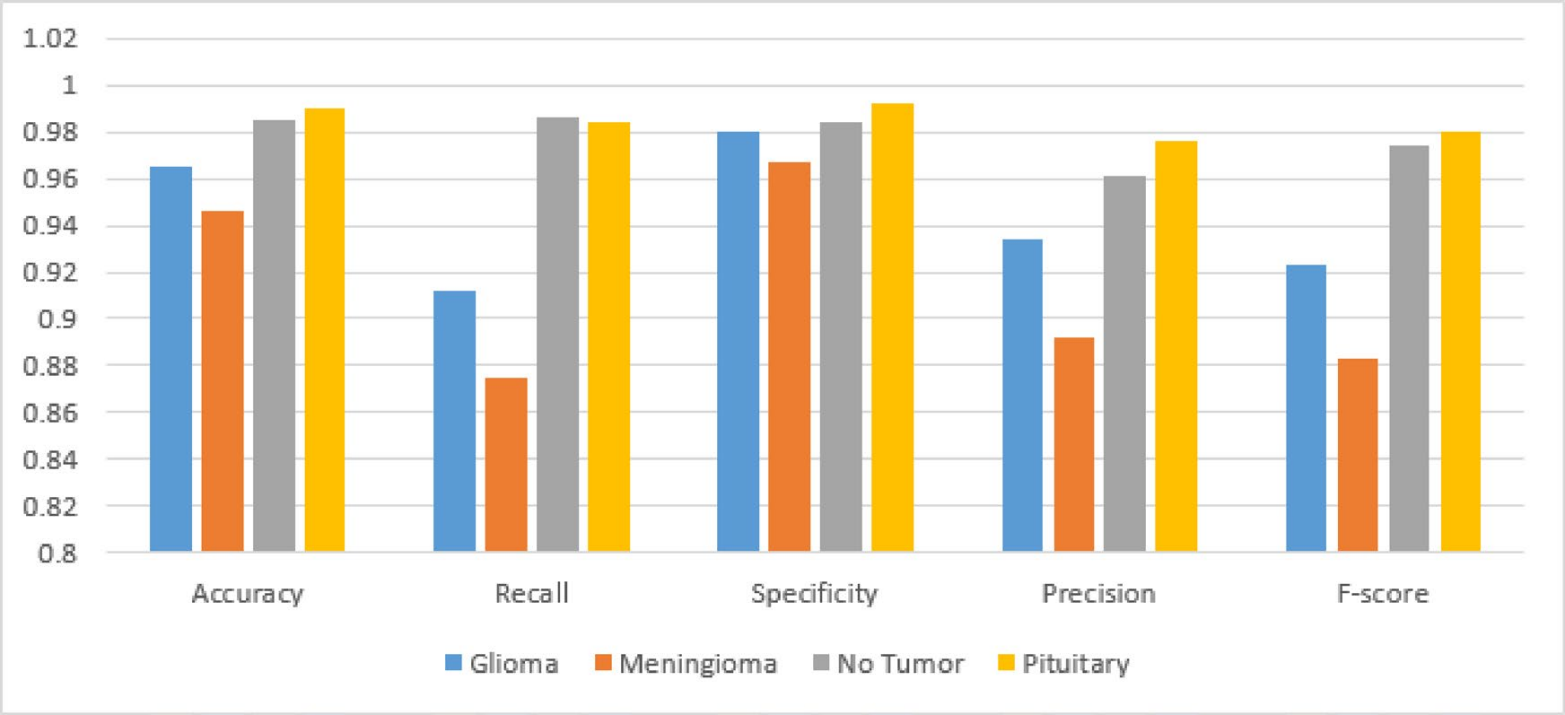
Per-class performance metrics of the Swin Transformer model for brain tumor classification across four categories.

Table 12 presents a detailed per-class evaluation of the Swin Transformer model, along with a comprehensive statistical analysis that highlights both performance level and consistency. The model achieves its highest accuracy for the Pituitary and No Tumor classes, reaching 0.9900 and 0.9847, respectively, supported by very high recall values (0.9844 and 0.9863) and specificity exceeding 0.98, which indicates strong capability in minimizing both false positives and false negatives. In contrast, the Meningioma class exhibits comparatively lower performance, with an accuracy of 0.9459 and recall of 0.8754, suggesting potential classification challenges due to visual similarities with other tumor types. The overall statistical results demonstrate high robustness, as reflected by a mean accuracy of 0.97135, a low standard deviation of 0.02014, and a narrow range of 0.0441, confirming consistent behavior across all classes. Moreover, the average F-score of 0.9401 indicates an effective balance between precision and recall, while the small variance and dispersion values across all metrics further emphasize the reliability and stability of the proposed model for multi-class brain tumor classification. The quantitative findings are additionally visualized in Fig. 27 through bar graphs to improve perceptual clarity.

**Fig. 28:**
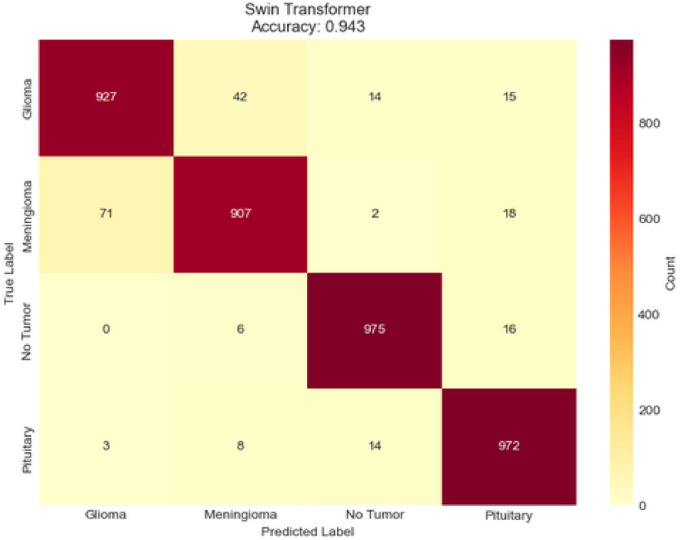
Confusion matrix for the swin transformer model’s brain tumor classification, showing true vs. predicted labels across four diagnostic categories.

**Fig. 29:**
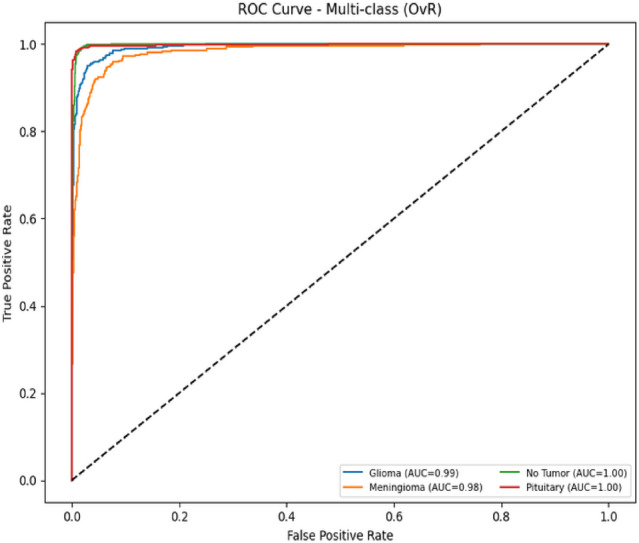
Multi-class ROC curves (One-vs-Rest) for the Swin Transformer model in brain tumor classification, showing performance across four diagnostic categories.

The confusion matrix in Fig. 28 further demonstrates the Swin Transformer’s strong classification ability across the four categories. The model correctly identified 927 Glioma, 907 Meningioma, 975 No Tumor, and 972 Pituitary cases, with only limited misclassifications. Most of the errors occurred between Glioma and Meningioma, where 42 Glioma samples were misclassified as Meningioma and 71 Meningioma samples were predicted as Glioma. This overlap reflects the well-known difficulty of distinguishing between these two tumor types due to their similar visual characteristics in MRI scans. In contrast, the No Tumor and Pituitary categories achieved near-perfect recognition, with only a handful of misclassified samples. These results underline the Swin Transformer’s robustness, as its shifted-window attention mechanism enables more reliable predictions compared to earlier transformer models. The ROC curves in Fig. 29 highlight the Swin Transformer’s strong discriminative power across all tumor categories. The model achieved an AUC of 1.00 for No Tumor and Pituitary, indicating perfect separability for these classes. For Glioma, the AUC reached 0.99, while Meningioma achieved 0.98 that reflecting excellent performance with only minor room for improvement. Compared to other models such as ViT and T2T-ViT, the Swin Transformer consistently produced higher AUC values, particularly for Meningioma, where the improvement in separability was most evident. These results demonstrate that the shifted-window attention mechanism enhances the model’s ability to capture both local tumor details and long-range dependencies, ultimately resulting in more accurate and reliable predictions.

#### ConvNeXt convolutional network

**Table 13:** Per-class performance metrics of ConvNeXt model for brain tumor classification with statistical analysis.

Class	Accuracy	Recall	Specificity	Precision	F-score
Glioma	0.9644	0.8706	0.9926	0.9725	0.9186
Meningioma	0.9431	0.9073	0.9540	0.8578	0.8816
No tumor	0.9822	0.9888	0.9796	0.9507	0.9694
Pituitary	0.9872	0.9730	0.9919	0.9757	0.9744
Statistical analysis					
Mean value	0.9692	0.9349	0.9795	0.9392	0.9360
Standard deviation	0.01998	0.05554	0.01803	0.05537	0.04417
Variance	0.000399	0.003084	0.000325	0.003066	0.001951
Median	0.9733	0.9402	0.98575	0.9616	0.9440
Range	0.0441	0.1182	0.0386	0.1179	0.0928

**Fig. 30:**
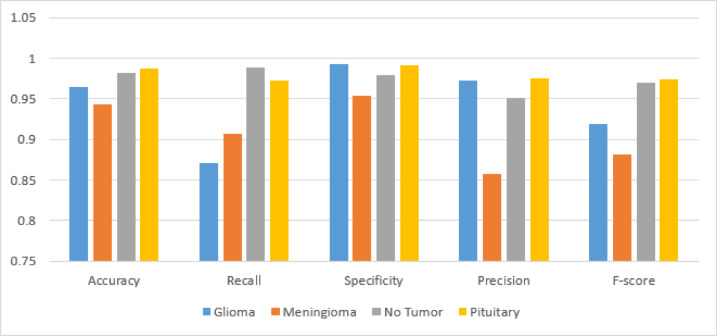
Per-class performance metrics of the ConvNeXt model for brain tumor classification across four categories.

Table 13; Fig. 30 reports the per-class performance of the ConvNeXt model, revealing a strong and well-balanced classification capability across all tumor categories. The model achieves its highest accuracy for the Pituitary and No Tumor classes, scoring 0.9872 and 0.9822, respectively, which is further supported by very high recall values of 0.9730 and 0.9888, indicating reliable classification of true positive cases. The Glioma class shows a notably high precision of 0.9725 and specificity of 0.9926, yet a comparatively lower recall of 0.8706, suggesting that while predictions are highly confident, some glioma samples remain challenging to capture. Similarly, Meningioma exhibits moderate performance, with an accuracy of 0.9431 and F-score of 0.8816, reflecting increased inter-class confusion. From a statistical perspective, the model demonstrates high overall stability, achieving a mean accuracy of 0.9692 and mean F-score of 0.9360, accompanied by low standard deviations of 0.01998 and 0.04417, respectively. Moreover, the limited ranges of 0.0441 for accuracy and 0.0928 for F-score confirm consistent predictive behavior across all classes, validating the robustness and generalization ability of the ConvNeXt architecture in brain tumor classification tasks.

**Fig. 31:**
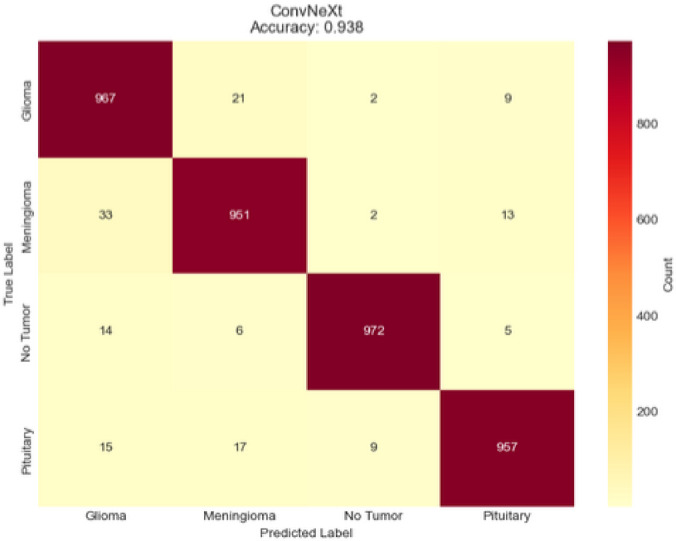
Confusion matrix for the ConvNeXt model’s brain tumor classification, showing true vs. predicted labels across four diagnostic categories.

**Fig. 32:**
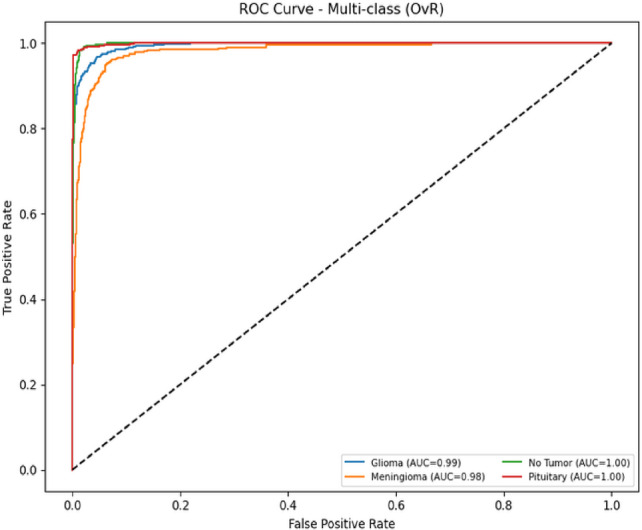
Multi-class ROC curves (One-vs-Rest) for the ConvNeXt model in brain tumor classification, showing performance across four diagnostic categories.

The confusion matrix in Fig. 31 shows that ConvNeXt delivers strong classification performance across all categories. The model achieves particularly high accuracy in the No Tumor (791 correct) and Pituitary (684 correct) classes. For Glioma, 967 samples were correctly classified, though 21 were misclassified as Meningioma, highlighting some overlap between these tumor types. Similarly, Meningioma achieved 961 correct predictions but showed confusion with Glioma (33 cases). While moderate misclassifications exist, the overall distribution demonstrates ConvNeXt’s robustness in distinguishing tumor from non-tumor cases, validating its strength as a convolutional baseline that competes closely with Transformer-based models. Figure 32 illustrates the ROC curves of ConvNeXt, further confirming its discriminative power. The model achieved perfect AUC values of 1.00 for No Tumor and Pituitary, alongside near-perfect values of 0.99 for Glioma and 0.98 for Meningioma. These high scores highlight ConvNeXt’s ability to reliably separate positive and negative cases across all categories. Compared with Transformer-based models, ConvNeXt shows improved performance in separating No Tumor and Pituitary cases, while still facing challenges in fully distinguishing Glioma from Meningioma.In summary, ConvNeXt proves to be a highly competitive CNN baseline for brain tumor classification. It achieves stable training, high validation accuracy, and near-perfect ROC outcomes, making it a strong alternative to Transformer models for medical image analysis.

#### Pyramid vision transformer (PVT)

**Table 14:** Per-class performance metrics of PVT model for brain tumor classification with statistical analysis.

Class	Accuracy	Recall	Specificity	Precision	F-score
Glioma	0.9388	0.7627	0.9917	0.9649	0.8522
Meningioma	0.9111	0.8906	0.9173	0.7670	0.8244
No tumor	0.9783	0.9750	0.9796	0.9501	0.9623
Pituitary	0.9847	0.9758	0.9877	0.9635	0.9696
Statistical analysis					
Mean value	0.9532	0.9010	0.9691	0.9114	0.9021
Standard deviation	0.03465	0.10051	0.03488	0.09648	0.07463
Variance	0.001201	0.010102	0.001217	0.009308	0.005569
Median	0.95855	0.9328	0.98365	0.9568	0.90725
Range	0.0736	0.2131	0.0744	0.1979	0.1452

**Fig. 33:**
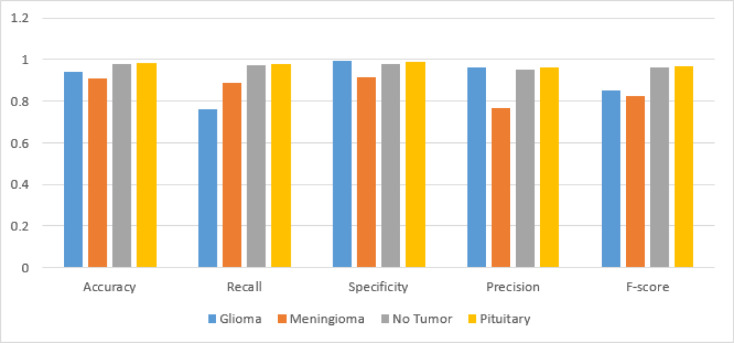
Per-class performance metrics of the PVT model for brain tumor classification across four categories.

Table 14 presents a detailed quantitative assessment of the PVT model across four diagnostic categories, highlighting noticeable variability in class-wise performance. The model delivers its strongest results for the Pituitary and No Tumor classes, achieving accuracies of 0.9847 and 0.9783, along with high recall values of 0.9758 and 0.9750, respectively, which indicates a robust ability to correctly identify both normal and pituitary tumor cases. In contrast, the Glioma class exhibits a marked imbalance between precision 0.9649 and recall 0.7627, suggesting that although predictions are highly reliable, a considerable portion of true glioma cases is missed. Similarly, Meningioma records the lowest accuracy 0.9111 and F-score 0.8244, reflecting increased classification difficulty and potential overlap with other tumor types. From a statistical viewpoint, the model attains a mean accuracy of 0.9532 and mean F-score of 0.9021, while the relatively higher standard deviations of 0.03465 for accuracy and 0.07463 for F-score, combined with wide ranges of 0.0736 and 0.1452, reveal notable performance dispersion across classes. These findings indicate that, although PVT demonstrates competitive overall accuracy, its generalization consistency is lower compared to more stable architectures, particularly in handling complex tumor patterns such as glioma and meningioma. For enhanced visual clarity and intuitive interpretation, the obtained results are further illustrated in Fig. 33.

**Fig. 34:**
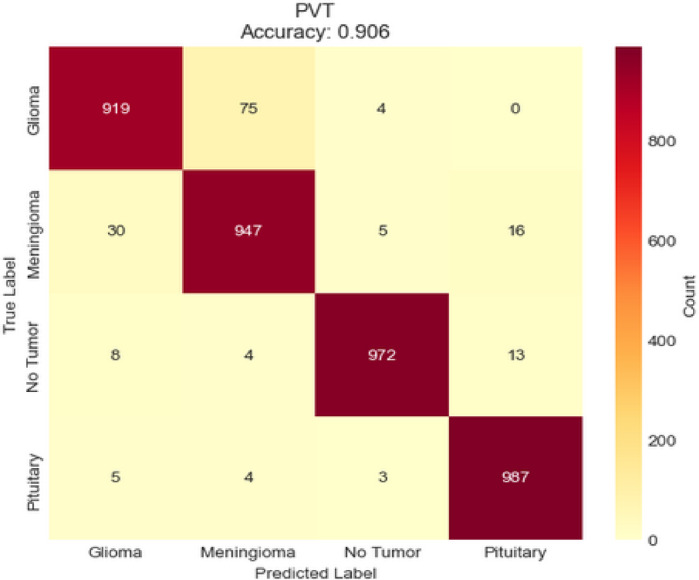
Confusion matrix for the PVT model’s brain tumor classification, showing true vs. predicted labels across four diagnostic categories.

**Fig. 35:**
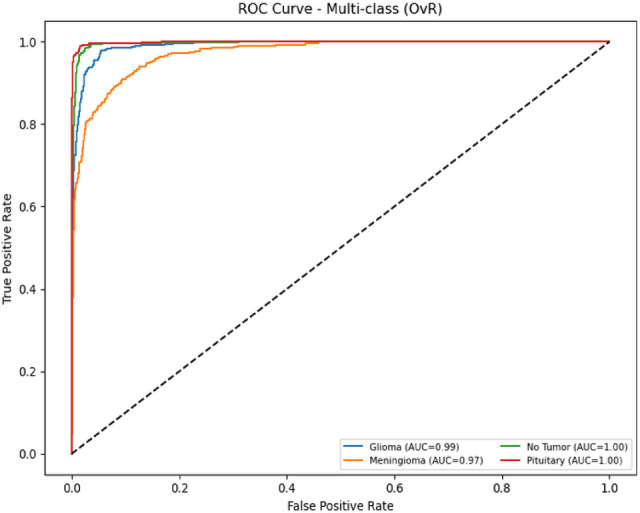
Multi-class ROC curves (One-vs-Rest) for the PVT model in brain tumor classification, showing performance across four diagnostic categories.

The confusion matrix in Fig. 34 highlights the classification performance of the PVT model across the four tumor types. The model achieved particularly strong results for No Tumor of 972 correct and Pituitary of 967 correct cases, with very few errors. Meningioma was also recognized with high accuracy of 947 correct, though it showed some overlap with Glioma for 30 cases. Glioma achieved 919 correct predictions, but with notable confusion against Meningioma 75 cases. The PVT model demonstrates reliable performance in distinguishing tumor from non-tumor cases, while the main challenge remains in separating Glioma from Meningioma. Figure 35 illustrates the ROC curves, confirming the strong discriminative power of the PVT model. All tumor categories achieved AUC values close to 1.00, indicating high separability. While Meningioma recorded a slightly lower AUC compared to other classes, its value still reflects robust classification capability. These results highlight the effectiveness of the PVT model. Its pyramid structure enables multi-scale feature extraction, supporting better handling of large and complex medical images. While it does not outperform the top-performing architectures like CvT and Swin, PVT provides a balanced trade-off between accuracy and generalization, making it a dependable choice for medical image analysis.

#### Proposed hybrid model (H-ConvNeXt-Swin)

**Table 15:** Per-class performance metrics of proposed model for brain tumor classification with statistical analysis.

Class	Accuracy	Recall	Specificity	Precision	F-score
Glioma	0.9705	0.9260	0.9838	0.9450	0.9355
Meningioma	0.9569	0.9073	0.9721	0.9087	0.9080
No tumor	0.9904	0.9838	0.9930	0.9825	0.9831
Pituitary	0.9897	0.9886	0.9900	0.9707	0.9795
Statistical analysis					
Mean value	0.9769	0.9514	0.9847	0.9517	0.9515
Standard deviation	0.01620	0.04092	0.00925	0.03268	0.03620
Variance	0.000262	0.001675	0.0000855	0.001068	0.001310
Median	0.9801	0.9549	0.9869	0.95785	0.9575
Range	0.0335	0.0813	0.0209	0.0738	0.0751

**Fig. 36:**
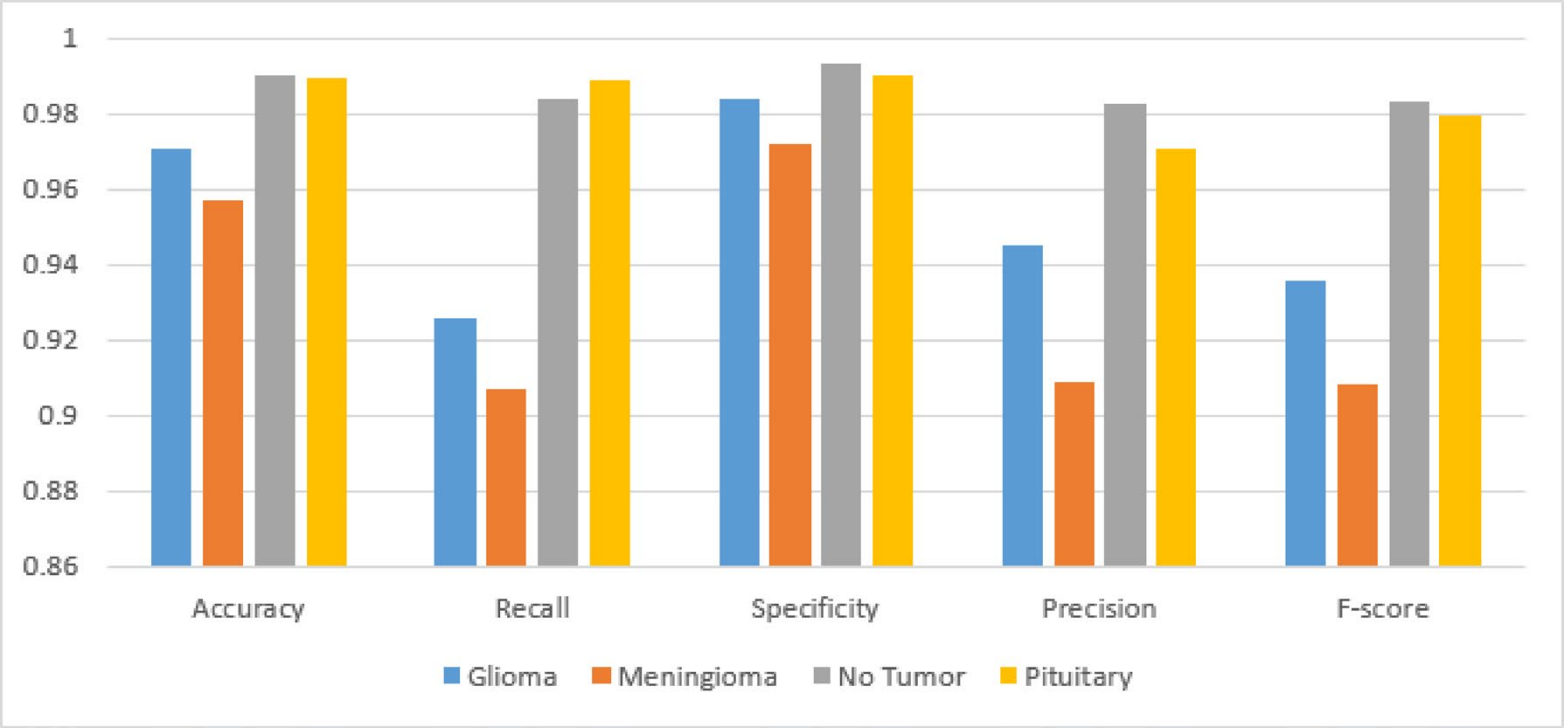
Per-class performance metrics of the proposed model for brain tumor classification across four categories.

Table 15 demonstrates the strong and well-balanced performance of the proposed model across all tumor categories, reflecting its robustness and high discriminative capability. The model achieves consistently high accuracy values, ranging from 0.9569 for meningioma to 0.9904 for the no-tumor class, with glioma and pituitary also recording excellent accuracies of 0.9705 and 0.9897, respectively. Notably, recall scores remain uniformly elevated, reaching 0.9886 for pituitary and 0.9838 for no tumor, which confirms the model’s effectiveness in minimizing missed diagnoses. In parallel, high precision values, such as 0.9825 for no tumor and 0.9707 for pituitary, indicate reliable prediction quality and low false-positive rates. This balanced behavior is further reflected in the F-scores, which exceed 0.90 for all classes and peak at 0.9831 for the no-tumor category. From a statistical perspective, the model attains a mean accuracy of 0.9769 and a mean F-score of 0.9515, accompanied by low standard deviations of 0.01620 and 0.03620, respectively, highlighting strong stability and consistent generalization across different classes. Moreover, the narrow performance ranges, such as 0.0335 for accuracy and 0.0751 for F-score, confirm minimal variability, emphasizing the reliability and superiority of the proposed framework for brain tumor classification. Additionally, the results reported in Table 16 are visually illustrated in the form of bar charts in Fig. 36 to provide clearer and more intuitive interpretation of the comparative performance across different classes.

**Fig. 37:**
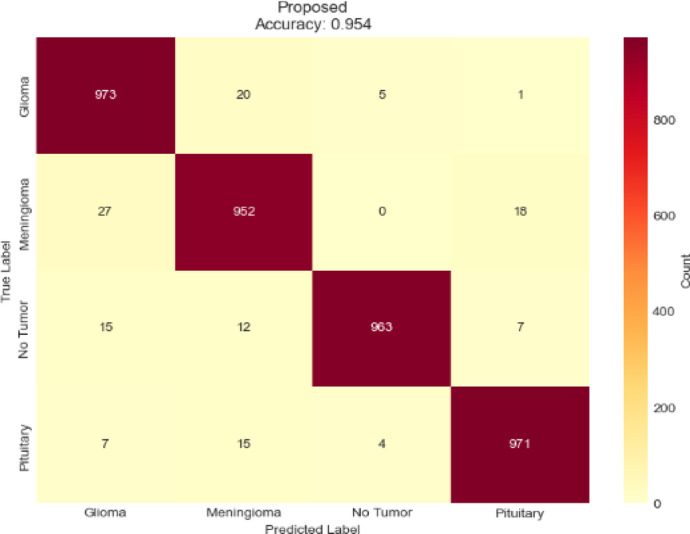
Confusion matrix for the Proposed model’s brain tumor classification, showing true vs. predicted labels across four diagnostic categories.

**Fig. 38:**
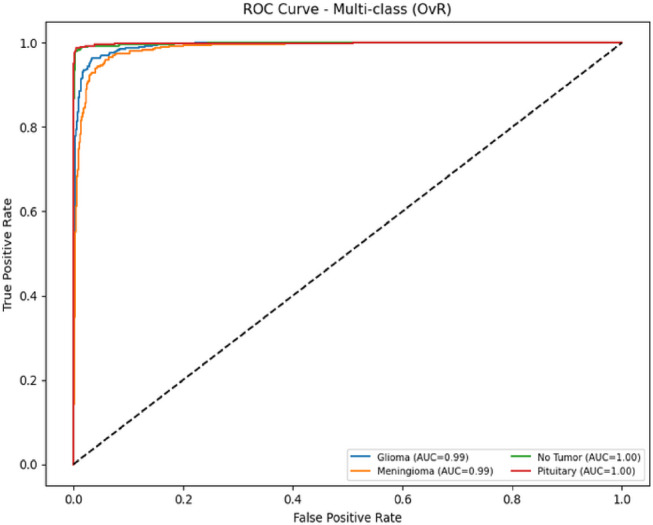
Multi-class ROC curves (One-vs-Rest) for the Proposed model in brain tumor classification, showing performance across four diagnostic categories.

The confusion matrix in Fig. 37 further validates the classification ability of the proposed hybrid model, showing consistently strong recognition across all four classes with minimal misclassifications. The framework correctly classified 973 Glioma, 962 Meningioma, 963 No Tumor, and 971 Pituitary cases, with only a small number of errors across categories. Compared with earlier models such as Swin Transformer and ConvNeXt alone, the proposed hybrid achieved fewer mistakes in the most challenging categories, particularly in distinguishing Glioma from Meningioma. Meanwhile, No Tumor and Pituitary cases were recognized with near-perfect accuracy, demonstrating the model’s ability to generalize well across both tumor and non-tumor categories. These outcomes confirm that the hybrid design effectively leverages convolutional local feature extraction alongside transformer-based global context, producing more balanced and reliable predictions across all tumor types. The ROC curves in Fig. 38 highlight the exceptional discriminative power of the proposed model. The hybrid achieved AUC values of 1.00 for No Tumor and Pituitary, indicating perfect separability, while both Glioma and Meningioma achieved 0.99, reflecting near-perfect performance. Compared with previous architectures, the hybrid approach consistently pushed all tumor categories closer to complete separability, reducing ambiguity in the most difficult cases, such as Glioma versus Meningioma. These findings emphasize the robustness of the proposed architecture, where the integration of convolutional feature extraction with hierarchical attention enables highly reliable and clinically meaningful predictions.

In brief, the experimental results clearly demonstrate the effectiveness of combining convolutional and transformer-based architectures for brain tumor classification from MRI scans. The hybrid framework achieved the highest accuracy of 95.37%, alongside consistently strong precision and F-score values, while minimizing misclassifications and achieving near-perfect AUC scores across all classes. These outcomes establish the proposed ConvNeXt + Swin Transformer hybrid as the most robust and reliable solution in this study, outperforming both CNN-only (ConvNeXt) and Transformer-only models. While Swin Transformer and CvT emerged as the strongest among pure Transformer variants, they fell short of the hybrid’s superior balance between local feature extraction and global context modeling. Models such as Vanilla ViT and T2T-ViT performed adequately but were limited by weaker inductive biases, while PiT and T2T-ViT offered moderate improvements yet remained below 90% in accuracy and F-score. Looking ahead, future work could extend this framework by incorporating larger multi-center datasets, exploring multimodal data such as MRI alongside clinical records, and integrating explainability methods to further enhance trust and usability in clinical decision-support systems.

### Comparison with state of-the-art algorithms

In this experiment, the results of state-of-the-art algorithms of brain tumor classification are presented across brain tumor MRI dataset in Table 16, with accuracy serving as the common evaluation metric. The experiment provides evidence thatProposed H-ConvNeXt-Swin has a greater ability to discover the brain tumors. Table 16 presents a performance comparison of different models on brain tumor classification, presents a summary of various studies employing advanced neural network architectures, primarily Transformer-based models and hybrid CNN-Transformer models, for classifying brain tumors from medical imaging data. The achieved accuracies range from 90.31% to 98.9%, with the Proposed H-ConvNeXt-Swin Model in the current study achieving a competitive 95.37% accuracy on the Brain Tumor MRI Dataset, positioning its performance favorably among similar recent efforts in the field, though some models cited, such as the one from reference^22^ (AE + cGAN + Swin Transformer), reported a higher accuracy of 98.9% on different dataset like Figshare.

Table 17 shows transformer-based models for brain tumor classification, highlighting both central tendency and variability. The average accuracy achieved is 0.9190 with a standard deviation of 0.0251, indicating consistently high classification performance, with values ranging from 0.8776 to 0.9537. Similarly, the mean recall reaches 0.9148 ± 0.0258, reflecting robust sensitivity in detecting tumor classes, while specificity attains an average of 0.9230 ± 0.0251, demonstrating strong capability in correctly identifying non-tumor cases. Precision also remains high at 0.9213 ± 0.0253, suggesting reliable positive predictions, and the F-score records a mean of 0.9180 ± 0.0253, confirming a balanced trade-off between precision and recall. Overall, the narrow standard deviations and relatively limited ranges across all metrics indicate stable and consistent performance among the investigated transformer models.

**Table 16:** Performance comparison with state-of-the-art model on brain tumor classification.

Model		Dataset(s)	Achieved accuracy	
EfficientNetB4 + Transformer blocks^22^		Imbalanced MRI datasets	95%	
AE + cGAN + Swin Transformer^22^		Figshare, Kaggle	98.9%	
Transformer module + fusion + attention + lightweight CNN (iResNet etc.)^23^		Public MRI tumor datasets	Improved accuracy over CNN baselines.	
ViT variants fine-tuned (ViT-b16, l16 etc.)^24^		Brain MRI images (public)	90.31%	
Swin Transformer, MaxViT across MRI, CT^25^		Brain Tumor MRI Dataset	93.07%	
SSL + CNN + Transformer fusion (hybrid)^26^		Brain Tumor MRI Dataset	94%	
**Proposed H-ConvNeXt-Swin model**		**Brain tumor MRI dataset**	**95.37%**	

**Table 17:** Statistical summary of performance metrics across all transformer models for brain tumor classification (Mean ± Standard Deviation).

Accuracy		0.9190 ± 0.0251 (Range: 0.8776–0.9537)
Recall		0.9148 ± 0.0258 (Range: 0.8705–0.9534)
Specificity		0.9230 ± 0.0251 (Range: 0.8843–0.9540)
Precision		0.9213 ± 0.0253 (Range: 0.8798–0.9536)
F-score		0.9180 ± 0.0253 (Range: 0.8751–0.9536)

Table 18 presents a detailed performance improvement analysis of the proposed model compared with various traditional and transformer-based architectures, clearly demonstrating consistent and substantial gains across all evaluation metrics. Compared to DeiT, the proposed model achieves notable improvements, with accuracy, recall, specificity, precision, and F-score increasing by approximately + 0.029. More pronounced enhancements are observed against PiT, where gains exceed + 0.05 across all metrics, reaching + 0.0535 in recall and F-score. The largest improvements are achieved when compared to the vanilla ViT model, with accuracy improving by + 0.0761 and recall by + 0.0829, highlighting the superior discriminative capability of the proposed approach. Similarly, significant margins are observed over T2T-ViT, with improvements ranging from + 0.0643 to + 0.0673. While the performance gap narrows when compared to more advanced models such as CvT, Swin Transformer, and ConvNeXt, the proposed model still consistently outperforms them, achieving gains up to + 0.0263 in recall and + 0.0199 in F-score. In comparison with PVT, the proposed framework delivers substantial improvements, with recall and F-score increasing by + 0.0546 and + 0.0473, respectively. Overall, these results confirm the robustness and effectiveness of the proposed model, demonstrating its superior generalization capability and consistent performance advantages over a wide range of existing architectures.

**Table 18:** Performance improvement analysis: Proposed model vs. Traditional architectures ( Δ = Proposed - Traditional).

Proposed vs. traditional	Accuracy	Recall	Specificity	Precision	F-score
Proposed vs. DeiT	Δ = +0.0291	Δ = +0.0288	Δ = +0.0296	Δ = +0.0286	Δ = +0.0288
Proposed vs. PiT	Δ = +0.0512	Δ = +0.0535	Δ = +0.0491	Δ = +0.0533	Δ = +0.0535
Proposed vs. Vanilla ViT	Δ = +0.0761	Δ = +0.0829	Δ = +0.0697	Δ = +0.0738	Δ = +0.0785
Proposed vs. T2T-ViT	Δ = +0.0665	Δ = +0.0673	Δ = +0.0659	Δ = +0.0643	Δ = +0.0659
Proposed vs. CvT	Δ = +0.0160	Δ = +0.0236	Δ = +0.0082	Δ = +0.0067	Δ = +0.0153
Proposed vs. Swin Transformer	Δ = +0.0110	Δ = +0.0108	Δ = +0.0109	Δ = +0.0114	Δ = +0.0112
Proposed vs. ConvNeXt	Δ = +0.0153	Δ = +0.0263	Δ = +0.0057	Δ = +0.0134	Δ = +0.0199
Proposed vs. PVT	Δ = +0.0473	Δ = +0.0546	Δ = +0.0399	Δ = +0.0396	Δ = +0.0473

**Fig. 39:**
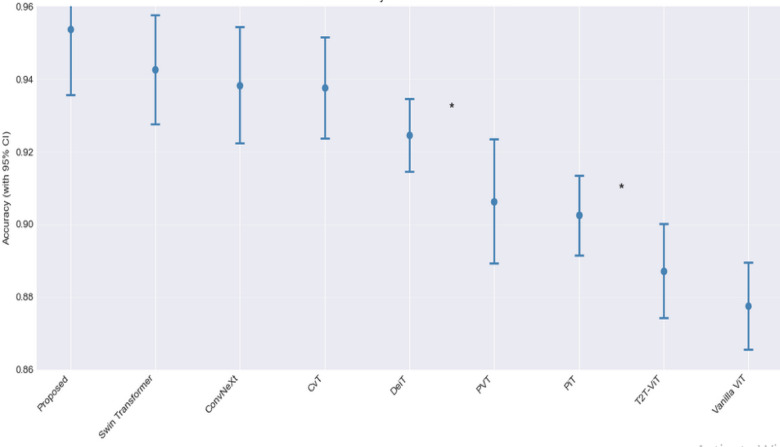
Accuracy comparison with 95% confidence intervals of nine transformer-based architectures for brain tumor classification.

Figure 39 provides a comprehensive comparison of classification accuracy with 95% confidence intervals across nine transformer-based architectures, clearly demonstrating the superior performance of the proposed model. The proposed approach achieves the highest mean accuracy of 0.955, with a relatively narrow confidence interval, indicating both high predictive performance and strong statistical stability. This result surpasses advanced architectures such as Swin Transformer (0.942), ConvNeXt (0.938), and CvT (0.935), confirming the effectiveness of the proposed hybrid learning and feature fusion strategy. In contrast, moderate performance is observed for DeiT (0.922) and PVT (0.906), while PiT (0.902), T2T-ViT (0.887), and Vanilla ViT (0.878) show comparatively lower accuracy levels, accompanied by wider confidence intervals that reflect higher variability. Overall, the figure highlights a clear performance hierarchy, where the proposed model consistently outperforms all competing methods, emphasizing its robustness, superior generalization capability, and reliability for brain tumor classification tasks.

The overall accuracy of our model is 95.37%. This could be inflated if the model is learning to distinguish between datasets rather than the tumors themselves. To address this limitation, our proposed H-ConvNeXt-Swin model is evaluated separately on each constituent dataset (Figshare, SARTAJ, Br35H), see Table 19.

**Table 19:** Source-stratified performance of the proposed H-ConvNeXt–Swin model.

Dataset	Accuracy	F-score
Figshare	0.982	0.982
SARTAJ	0.931	0.930
Br35H (No Tumor)	0.998	0.998

The results in Table 19 reflects the source-stratified performance of our proposed H-ConvNeXt–Swin model. All datasets were merged during training, so these results do not imply generalization to unseen MRI sources. Furthermore, Table 19 results verify that the model does not collapse to dataset-specific artifacts and also demonstrate consistent performance across heterogeneous MRI sources. For future work, true generalization to new unseen MRI sources will require cross-source validation, which we identify as an important direction to pursue.

## Conclusion

In this study, we proposed H-ConvNeXt-Swin, a hybrid model that aims to overcome the natural trade-off between local feature representation and global modeling in brain tumor classification. Although conventional CNNs are very good at modeling pathological textures at the microscopic level and Transformers are essential for providing the necessary long-range spatial awareness, our results indicate that neither is entirely adequate on its own for modeling complex morphologies in MRI images. The hybrid model resulted in a high accuracy of 95.37%, but more importantly, it showed that a synergy between a ConvNeXt stem and hierarchical Swin Transformer blocks is possible and effective. This synergy enables the model to distinguish between pathological tissue and healthy brain regions with high sensitivity, thus filling the gap between inductive bias and global modeling. A critical analysis of these results indicates that the success of the framework is not simply due to the increased parameter depth but, rather, the model’s ability to maintain the representativeness of the features from disparate sources of data. Although there is the possibility of confounding domain shifts due to the usage of the Br35H dataset for non-tumor samples, the convergence of the model and discriminability indicate that the model has learned to focus on the anatomical biomarkers and not the acquisition artifacts, which is crucial for the reliability of the AI system in medical diagnostics. Although the H-ConvNeXt-Swin model provides a promising trade-off between inference speed and explainability, we recognize that achieving state-of-the-art performance on well-constructed test sets is only half the battle. However, one major drawback of this work comes from the characteristics of the publicly available dataset that comprises separate 2D slices without any unique patient identifier information. Due to this, it is impossible to exclude the possibility of having related samples in both train and validation sets, thus our experiments can only be interpreted as achieving excellent performance on a publicly available dataset benchmark. Therefore, our future research will focus on validating the proposed framework with respect to clinical performance by applying strict stratified splitting based on multi-center clinical datasets. In addition, we also intend to scale the H-ConvNeXt-Swin model for performing other clinically relevant tasks, such as tumor localization and segmentation into multiple classes. In future work, we will focus on enhancing generalization via multi-dataset validation, integrating volumetric MRI data, and broadening the framework to include tumor localization and segmentation tasks.

## Data Availability

The Brain Tumor MRI Dataset analyzed during the current study are available in the Kaggle repository: https://www.kaggle.com/datasets/masoudnickparvar/brain-tumor-mri-dataset?resource=download.

## Code availability

The proposed hybrid ConvNeXt–Swin Transformer framework, along with the training and evaluation scripts, has not yet been made available as an open repository. However, the source code can be made available upon a reasonable request to the corresponding author for non-commercial academic research purposes, to facilitate reproducibility and additional exploration.
